# Structured missingness in biomedical research

**DOI:** 10.1038/s44488-026-00025-9

**Published:** 2026-10-09

**Authors:** Barbora Rehák Bučková, Charlotte Fraza, Cecilie Koldbæk Lemvigh, Camilla Bärthel Flaaten, Linn Sofie Sæther, Giulia Cattarinussi, Karen Marie Sandø Ambrosen, Ole Andreas Andreassen, Lars Tjelta Westlye, Christian Beckmann, Bjørn Hylsebeck Ebdrup, Paola Dazzan, Torill Ueland, Robin Mitra, Andre Marquand

**Affiliations:** 1 The Department of Medical Neuroscience of Radboud University Medical Centre, Nijmegen, The Netherlands; 2 Donders Institute for Brain, Cognition and Behaviour, Radboud University Nijmegen, Nijmegen, The Netherlands; 3 Center for Neuropsychiatric Schizophrenia Research and Center for Clinical Intervention and Neuropsychiatric Schizophrenia Research, Mental Health Centre Glostrup, Copenhagen University Hospital - Mental Health Services CPH, Copenhagen, Denmark; 4 Section for Clinical Psychosis Research, Division of Mental Health and Addiction, Oslo University Hospital, Oslo, Norway; 5 Division of Mental Health and Addiction, Centre for Precision Psychiatry, Institute of Clinical Medicine, University of Oslo, Oslo, Norway; 6 Department of Psychological Medicine, Institute of Psychiatry, Psychology and Neuroscience, King’s College London, London, UK; 7 Section for Precision Psychiatry, Division of Mental Health and Addiction, Oslo University Hospital, and University of Oslo, Oslo, Norway; 8 Department of Psychology, University of Oslo, Oslo, Norway; 9 Department of Clinical Medicine, Faculty of Health and Medical Sciences, University of Copenhagen, Copenhagen, Denmark; 10 National Institute for Health and Care Research (NIHR) Maudsley Biomedical Research Centre at South London and Maudsley NHS Foundation Trust and King’s College London, London, UK; 11 Department of Statistical Science, University College London, London, UK

**Keywords:** Computational biology and bioinformatics, Health care, Mathematics and computing, Medical research

## Abstract

Missing data are ubiquitous in biomedical research, and become particularly problematic when combining data from multiple clinical studies, an increasingly common practice for building models that generalise across populations. This process introduces complex structured patterns of missing data (’structured missingness’), where one study may omit variables that another collects, leaving deterministic gaps that standard imputation methods were not designed to handle. Here, we show that many popular imputation algorithms systematically fail under structured missingness, distorting the underlying statistical properties of the data in ways that conventional accuracy metrics cannot detect. Methods designed to preserve data distributions are more robust, and a hierarchical modelling approach that explicitly accounts for site-specific differences further improves performance. We introduce a multi-metric evaluation framework and a practical decision guide to support method selection. Our findings highlight the need for more principled imputation approaches in multi-site biomedical research and provide concrete tools to address this challenge.

## Introduction

**Fig. 1:**
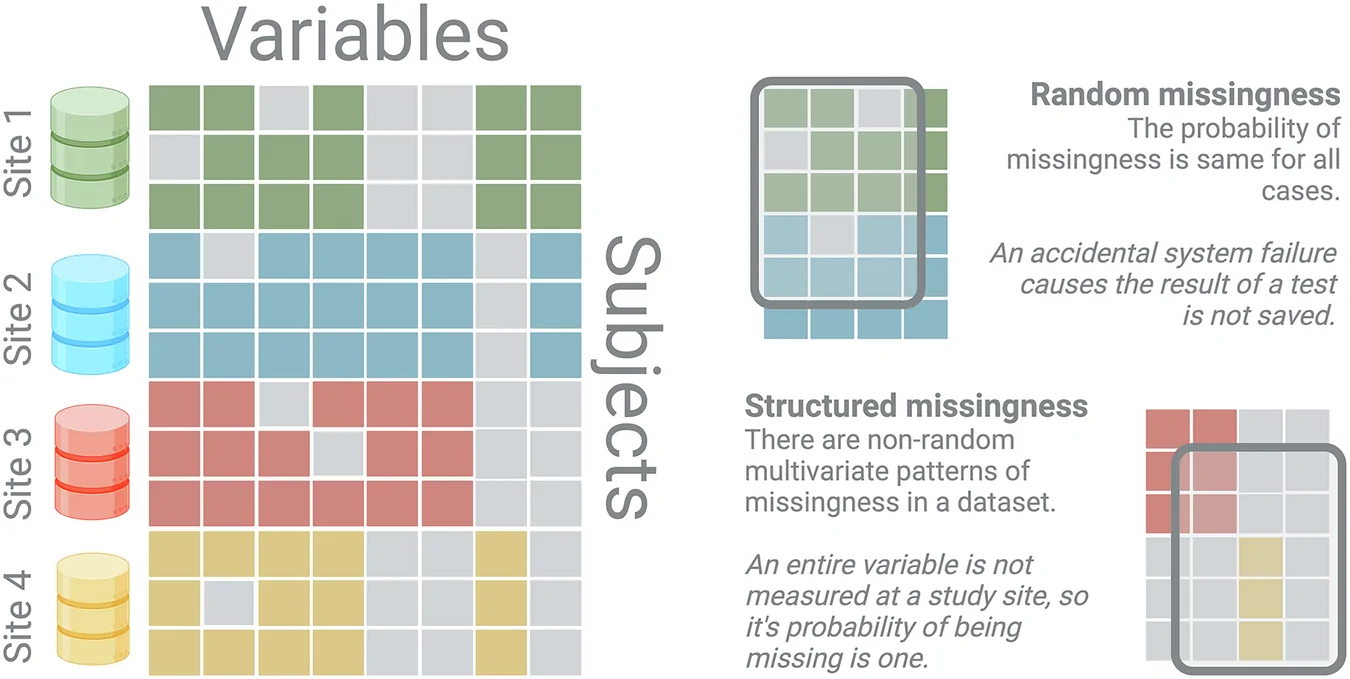
Illustration of structured and random missingness in a dataset. Certain variables are systematically unmeasured at specific study sites, leading to deterministic (probability of missingness equals one) blocks of missing data. This block missingness contrasts with random missingness, which occurs sporadically across the dataset. Created in BioRender. Rehak Buckova, B. (2026) https://BioRender.com/if0dtjs.

Widespread pooling of legacy datasets has revolutionised the pace of discovery in biomedical research^1–3^, increasing statistical power, improving demographic representation, and enabling more sophisticated machine learning (ML) applications^4–6^. However, a critical downside to cohort aggregation is that datasets collected under different protocols and for distinct research purposes rarely align perfectly in terms of measured variables, participant inclusion, and data completeness. This misalignment results in complex patterns of missingness, collectively known as a type of structured missingness^7^ (Fig. 1), a challenge that is ubiquitous in biomedical research, yet has not been systematically addressed.

In this article, we introduce a framework for handling structured missingness that is most specifically targeted towards ML and artificial intelligence (AI) applications, which are becoming increasingly indispensable tools for analysing large-scale biomedical datasets. In this context, structured missingness impedes the use of most ML/AI algorithms, which cannot natively handle missing values. The missing data problem has been extensively studied in classical inferential statistics, with methods like pattern mixture models^8^, full information maximum likelihood^9^, inverse probability weighting^10^, selection models^11^ and others providing principled solutions for group-level inference. However, these methods are not designed for out-of-sample prediction and do not produce completed datasets suitable as preprocessing steps for ML/AI pipelines, nor for individual subject inferences. Even algorithms that implement internal strategies for missingness, such as mean or zero substitution, missingness indicator variables in tree-based methods^12^, or learnt masking tokens in transformer-based architectures^13,14^, do not recover the underlying data distribution and introduce systematic bias in downstream analyses^15,16^. Proper imputation is therefore an essential and consequential preprocessing step for any ML/AI application to incomplete multi-site biomedical data.

Most classical imputation methods are still based on a framework developed by Rubin in the 1970s, which categorises missing data into three categories: missing completely at random, missing at random, and missing not at random^17–19^. Crucially, this framework conceptualises missingness as a probabilistic phenomenon, where any given data point has some non-zero chance of being observed. Although effective for many single-dataset scenarios^9,20–23^, it does not fully account for the systematic gaps introduced when datasets with non-overlapping variables are merged^24^.

An intuitive example is block missingness (also referred to as systematic missingness in the meta-analytical frameworks^25,26^) where data are absent due to study design rather than at random. For example, in clinical studies, symptom measures are by definition acquired in patients but not controls, resulting in deterministic gaps where entire sections of the data table are systematically missing. This deterministic nature (the probability of a control having a symptom scale measured is strictly zero) does not align with the probabilistic assumptions of classical frameworks. A ’missing not at random’ model, for instance, could handle a scenario where controls with certain subclinical traits might be less likely to be given a specific assessment, but it assumes their probability of being measured is still greater than zero. It is not equipped to handle the absolute certainty of block missingness, where the entire control group was systematically excluded by the study protocol. This deterministic structure, which often co-occurs with other patterns like dependency missingness or survey skip patterns^7^, means that the foundational assumptions of most imputation algorithms are violated, leaving their effectiveness on data with structured missingness largely unexplored^27^.

**Fig. 2:**
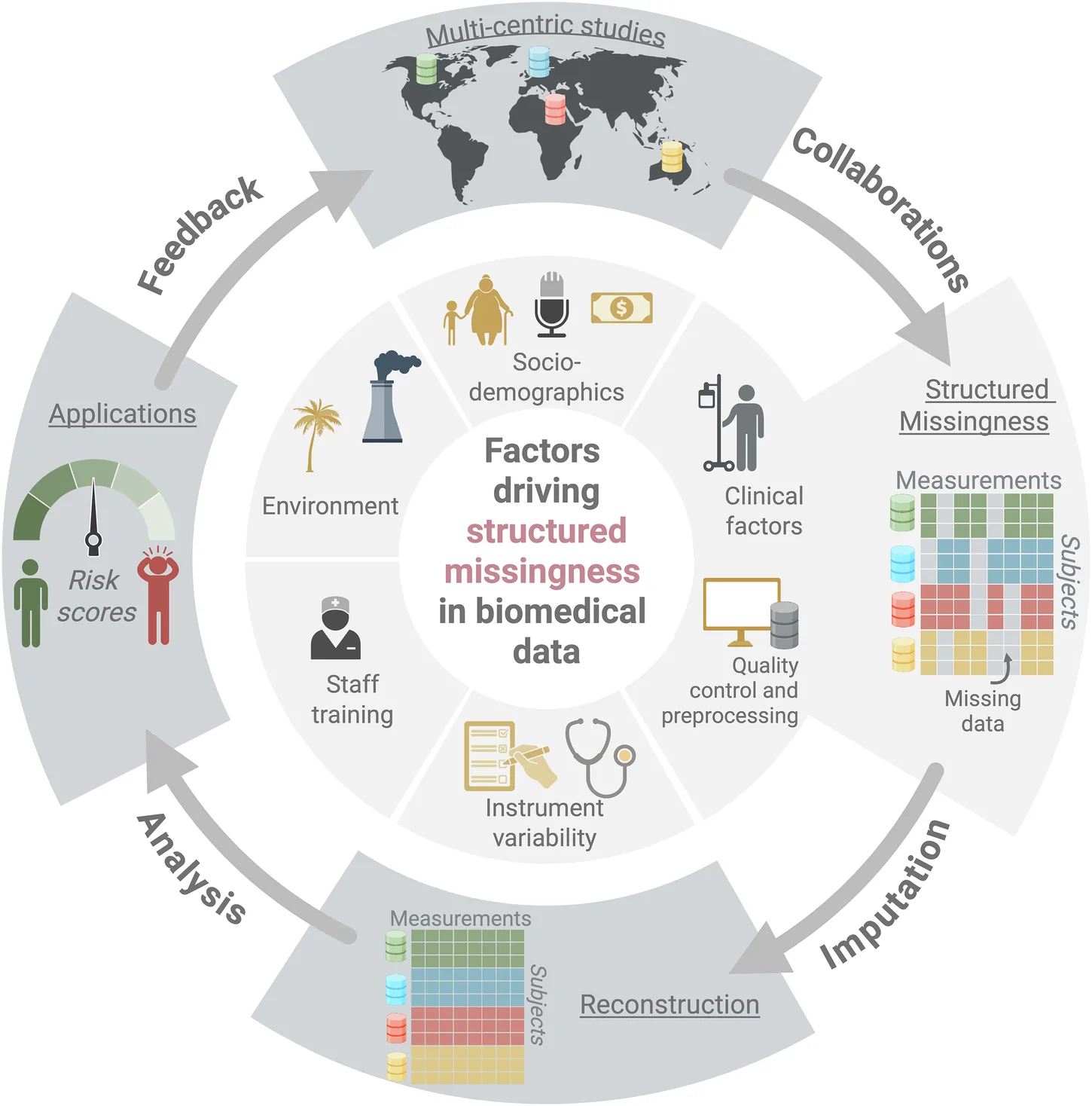
The cycle of collaboration and the factors driving structured missingness in biomedical data science. Collaborative research often involves merging databases from multiple centres, introducing structured missingness. Missingness may stem from differences in socio-demographic or clinical factors, site-specific data processing, variability introduced by medical devices, test administration, experiment protocols, staff training, or even environmental factors. Once datasets are merged, imputation, harmonisation or variable selection methods are applied to address missingness, ensuring a complete dataset for analysis. The quality of this step is critical, as it directly impacts downstream applications, such as predictive models for identifying patients at risk for certain medical conditions. Finally, the results of the research can further inform the acquisition of new measures, support further investigation and strengthen collaborations. Created in BioRender. Rehak Buckova, B. (2026) https://BioRender.com/9h65guv.

Another related, and equally critical issue in multi-site integration involves subtle differences in measurement techniques, which can introduce systematic inconsistencies and obscure biological or clinical insights (Fig. 2)^28^. For instance, multi-site clinical trials often measure outcomes such as quality of life^29^ or pain^30^ using different scales, which complicates direct comparisons. Even seemingly well-defined characteristics, such as race^31,32^, socioeconomic status^33^, or disease presence^28^, can differ across studies, cohorts, and socio-demographic context depending on the specific categories or cut-off values selected. Psychometric data can provide an even more nuanced example: despite the use of standardised constructs, different sites may assess cognition using distinct tests, each with unique scoring methods (i.e. having different distributions) and different sensitivity, specificity, and calibration to underlying latent traits, leading to results that are not comparable between instruments^34–39^. These issues can be further intensified by subtle changes in study protocols, recruitment strategies, test administration, language and cultural differences, and data processing pipelines.

To summarise, in this work, we propose a framework to address structured missingness that is rooted in an extensive simulation of realistic missingness patterns, robust methodologies for imputing data across diverse sources, and rigorous evaluation to ensure that integrated analyses yield reliable and interpretable insights. We show that widely used imputation methods, including Extra Trees and AutoComplete, fail systematically under structured missingness in ways that conventional accuracy metrics fail to detect, whereas donor-based approaches such as Multivariate Imputation by Chained Equations (MICE) and particularly its hierarchical extension, better preserve distributional and multivariate structure. Whilst this approach can be applied in many contexts, it is well suited towards ML and AI applications that require complete datasets. To achieve this, we: (i) conduct a large-scale simulation study to systematically assess the impact of structured missingness on the performance of state-of-the-art imputation algorithms; (ii) demonstrate that widely used imputation methods fail systematically under structured missingness and propose a comprehensive framework for evaluating imputation approaches. We introduce evaluation metrics that capture the complexities of structured missingness, moving beyond traditional single-dataset benchmarks to a multi-faceted assessment based on diverse statistical properties. Next, (iii) we apply the proposed evaluation approach to a large multi-site study on cognition in psychosis, focusing on the challenges of imputing structured missing data across multiple sites with different protocols and data collection practices. Finally, (iv) we discuss key considerations for real-world applications, including the integration of heterogeneous datasets with non-overlapping variables, and demonstrate how our approach improves imputation performance in a custom dataset of cognitive assessments.

## Results

### Simulation

**Fig. 3:**
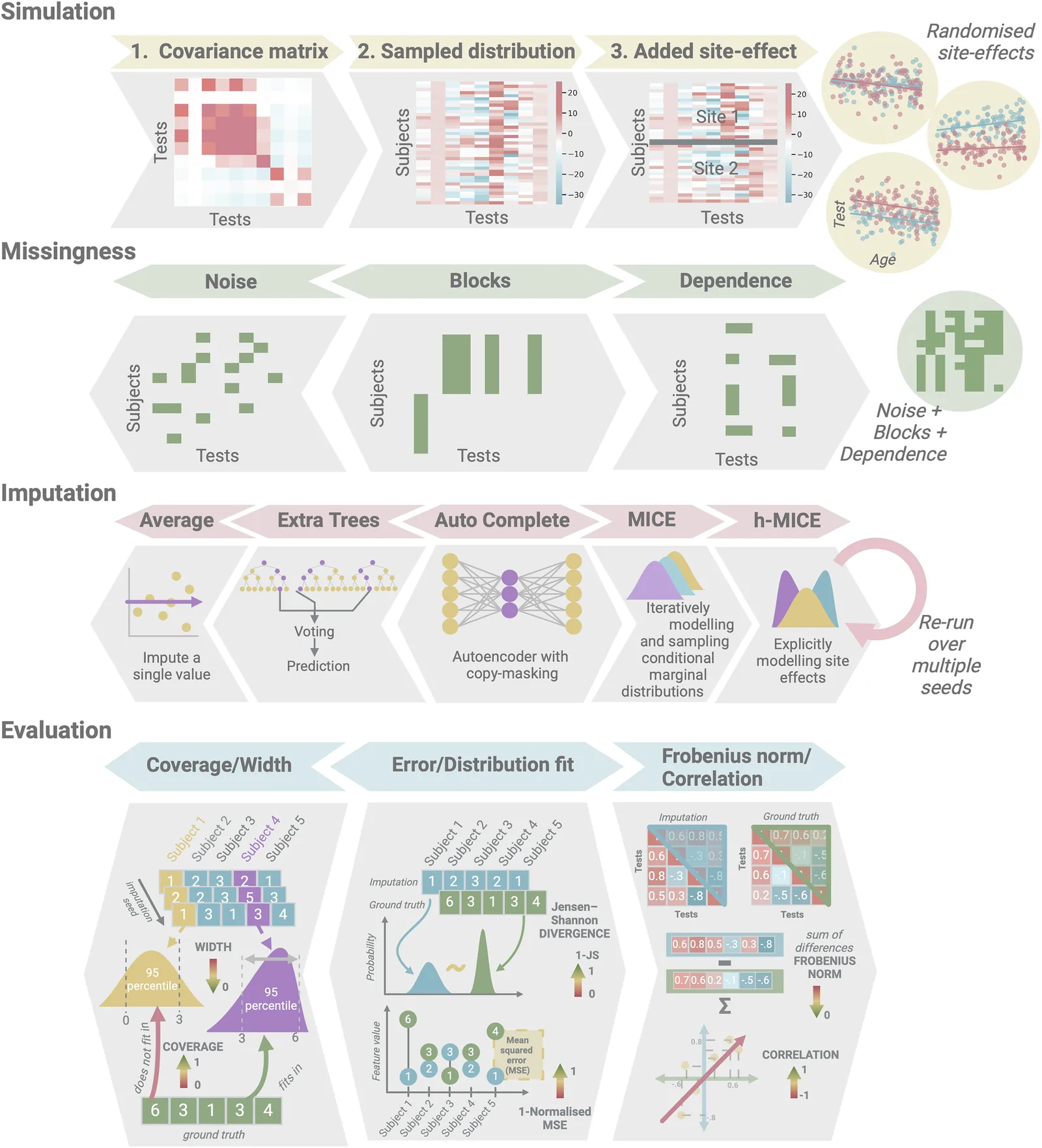
Simulation study pipeline. The first row illustrates the data generation process, starting with the extraction of a covariance matrix from a real-world dataset, generating synthetic datasets, and introducing site effects through random linear offsets (yellow circles). The second row depicts the three types of missingness introduced: missing completely at random, block missingness (entire variables deleted for a site), and dependence missingness (missingness in one variable driven by another). The missingness patterns were generated individually and in combination (green circle). The third row shows the imputation algorithms applied to the incomplete datasets: Average imputation, Extra Trees^41^, AutoComplete^42^, Multivariate Imputation by Chained Equations (MICE)^43^, and hierarchical MICE^44^, each of which was sampled 10 times. The final row shows the quality of imputation using subject-level metrics (coverage and width), variable-level metrics (1-normalised mean squared error (MSE) and 1-Jensen-Shannon divergence (JS)), and multivariable relationship metrics (Frobenius norm and upper-triangle correlation of correlation matrices). Created in BioRender. Rehak Buckova, B. (2026) https://BioRender.com/881ofvp.

To systematically assess the impact of structured missingness on imputation, we designed a simulation framework based on a realistic covariance structure extracted from a real-world cognitive dataset (Fig. 3). First, we extracted the covariance matrix from a real-world dataset of cognitive measures (see Simulation description in the ‘Methods’ section, or the GitHub repository^40^) to ensure realistic multivariate relationships. Using this as the basis for a multivariate normal distribution, we generated two synthetic datasets, each containing 40 variables, with sample sizes of either 50 or 500 subjects per site. To simulate site effects, we introduced random linear offsets across two predefined sites, with the magnitude of these offsets randomly varying between datasets and variables (for further details, see the ‘Methods’ section).

Next, we modelled three types of missingness: random missingness (a fixed proportion of values removed with no structure), block missingness (entire variables systematically absent at one site, reflecting non-overlapping measurement across sites), and dependency missingness (missingness in one variable conditional on another)-applied both individually and in combination. While this list is not exhaustive (see^24^ for a more comprehensive typology), it reflects several common scenarios encountered when merging heterogeneous datasets. In the case of block missingness, a key factor influencing the imputation quality is the degree of overlap between datasets. To assess this, we varied the number of shared variables between sites, testing overlaps of 4, 10, and 20 variables. Given the complexity of real-world missingness patterns, we simulated these mechanisms both individually and in combination. For technical details on the simulation process, we refer the reader to the ‘Methods’ section.

We compared five imputation approaches, each reflecting a distinct computational strategy: Extra Trees^41^, a tree-based ensemble method; AutoComplete^42^, an autoencoder-based deep learning approach; MICE^43^, which generates imputations via predictive mean matching; and a hierarchical extension of MICE, which explicitly models site-specific offsets for variables measured across multiple sites^44^. While the MICE approach produces multiple versions of the imputed dataset, which makes it possible to estimate the uncertainty, Extra Trees and AutoComplete lack this feature. To compensate for this limitation, both were run 10 times with distinct random seeds to approximate a distribution of imputed values. Finally, we benchmarked all methods against a simple average imputation. Although we do not recommend this approach, it is widely used in practice for its simplicity and computational efficiency, so we include it to illustrate its shortcomings and highlight the need for more advanced approaches.

Imputation procedures are often optimised and assessed using a single metric, such as mean squared error, which focuses solely on pointwise reconstruction accuracy^45^ and consequently can be misleading. For example, mean imputation may minimise the average error while distorting the underlying data distribution, thereby compromising downstream analyses^15,46^. This problem can be exacerbated in the presence of structured missingness, where blocks of missing data and site-specific effects make it nearly impossible to accurately estimate the magnitude and direction of bias. Consequently, methods focused solely on numerical accuracy may offer a biased and incomplete picture of the performance. To provide a more comprehensive assessment and identify criteria more robust towards structured missingness, we adopted a multi-dimensional evaluation framework (Table 1).

**Table 1:** Overview of imputation algorithms and evaluation metrics used in this study

Imputation algorithms		
Algorithm	Description	
Average Imputation	Replaces missing values with the mean of observed values. Simple, but introduces bias and distorts variability.	
Extra Trees	A tree-based ensemble method that selects features and splits nodes randomly. Reduces overfitting but requires sufficient data for stability^41^.	
AutoComplete	An autoencoder-based deep learning method that learns latent data structure to impute missing values. Effective for genetic data^42^.	
Multivariate Imputation by Chained Equations (MICE)	Iteratively models missing values using predictive mean matching. Flexible, but assumes correctly specified missingness mechanisms^43^.	
Hierarchical MICE	Extends MICE by modelling site-specific offsets for overlapping datasets. Improves accuracy in multi-cohort studies but requires sufficient subgroup data^44^.	

Evaluation methods				
Category of methods	Method	Description	Formula	Desired direction
Subject-level precision	Coverage	Does the true value lie within the imputed range?	$\frac{1}{{N}_{j}}\mathop{\sum }_{i = 1}^{{N}_{j}}{\mathbb{1}}({x}_{ij}\in [{\hat{q}}_{\alpha /2}^{(ij)},{\hat{q}}_{1-\alpha /2}^{(ij)}])$	More is better
	Width	How wide is the imputed range?	$\frac{1}{{N}_{j}}\mathop{\sum }_{i = 1}^{{N}_{j}}\frac{{U}_{ij}-{L}_{ij}}{\,{\mbox{SD}}\,({x}_{j})}$	Less is better
Variable-level accuracy	1-Normalised Mean Squared Error (1-NMSE)	How well are the original values reconstructed?	$1-\frac{\mathop{\sum }_{i = 1}^{{N}_{j}}{({x}_{ij}-{x}_{ij}^{(m)})}^{2}}{\mathop{\sum }_{i = 1}^{{N}_{j}}{({x}_{ij}-{\bar{x}}_{j})}^{2}}$	More is better
	1-Jensen-Shannon Divergence (1-JS)	How well are the original distributions preserved?	$1-\frac{D({p}_{j}\parallel {r}_{j})+D({q}_{j}\parallel {r}_{j})}{2}$	More is better
Multivariate relationships preservation	Frobenius norm	How much is the new correlation matrix different from the original one?	$\sqrt{{\sum }_{j,k}{({R}_{{\mbox{true}},jk}-{R}_{{\mbox{imp}},jk}^{(m)})}^{2}}$	Less is better
	Correlation	How much is the original one-to-one correlation preserved? (in terms of correlation)	$\,{\mbox{corr}}({\mbox{vec}}({R}_{{{{\rm{true}}}}}),{\mbox{vec}}({R}_{{\mbox{imp}}\,}^{(m)}))$	More is better

The table summarises the imputation algorithms, ranging from simple mean substitution to advanced machine learning and hierarchical methods, along with their descriptions. The evaluation metrics assess imputation quality in three domains: subject-level precision, variable-level accuracy, and preservation of multivariate relationships. For each metric, we provide a description, formula, and the desired performance direction. All metrics are computed exclusively on originally missing entries. Subject-level metrics (coverage, width) are averaged across subjects with missing values; variable-level metrics (1-NMSE, 1-JSD) are computed per variable and averaged across variables; multivariate metrics (Frobenius norm, correlation) are computed per imputation and averaged across imputations. *Parameter descriptions:i* indexes subjects, *j* indexes variables, *m* indexes imputations, and *N*_*j*_ is the number of subjects with missing values for variable *j*. In Coverage, *x*_*ij*_ is the true value for subject *i* and variable *j*, and $[{\hat{q}}_{\alpha /2}^{(ij)},{\hat{q}}_{1-\alpha /2}^{(ij)}]$ is the 95% percentile interval constructed across *m* = 10 imputed values ${\{{x}_{ij}^{(m)}\}}_{m = 1}^{M}$. In Width, *U*_*ij*_ and *L*_*ij*_ are the upper and lower bounds of the percentile interval, normalised by SD(*x*_*j*_), the standard deviation of variable *j*. In 1-Normalised MSE, ${x}_{ij}^{(m)}$ is the imputed value for subject *i*, variable *j* and imputation *m*, and ${\bar{x}}_{j}$ is the mean of the true values for variable *j*. In 1-Jensen-Shannon Divergence, *p*_*j*_ and *q*_*j*_ are the observed and imputed distributions for variable *j*, ${r}_{j}=\frac{1}{2}({p}_{j}+{q}_{j})$ is their average, and *D*(⋅∥⋅) is the Kullback-Leibler divergence. In Frobenius norm, *R*_true_ and ${R}_{\,{\mbox{imp}}\,}^{(m)}$ are the true and imputed correlation matrices for imputation *m*. In Correlation, corr(⋅,⋅) is the Pearson correlation between the vectorised upper triangles of *R*_true_ and ${R}_{\,{\mbox{imp}}\,}^{(m)}$ for imputation *m*.

We evaluated the algorithm at three complementary levels (Table 1). Subject-level uncertainty was assessed via *coverage*: the proportion of true values falling within the 95% interval across imputations, yielding one value per variable; and *interval width*: the range of imputed values across imputations and subjects for each variable, normalised by that variable’s standard deviation. Variable-level accuracy and distributional fidelity were calculated using *a 1-normalised mean squared error* (1-NMSE) and *1-Jensen-Shannon divergence* (1-JSD), both computed for each variable and each imputation. Finally, preservation of multivariate structure was assessed using the *Frobenius norm* and *correlation* of the upper triangles of the true and imputed correlation matrices, thus yielding one value per imputation. All metrics were computed exclusively on originally missing entries (full formal definitions in Table 1; full implementation and parameter details in ‘Methods’).

**Fig. 4:**
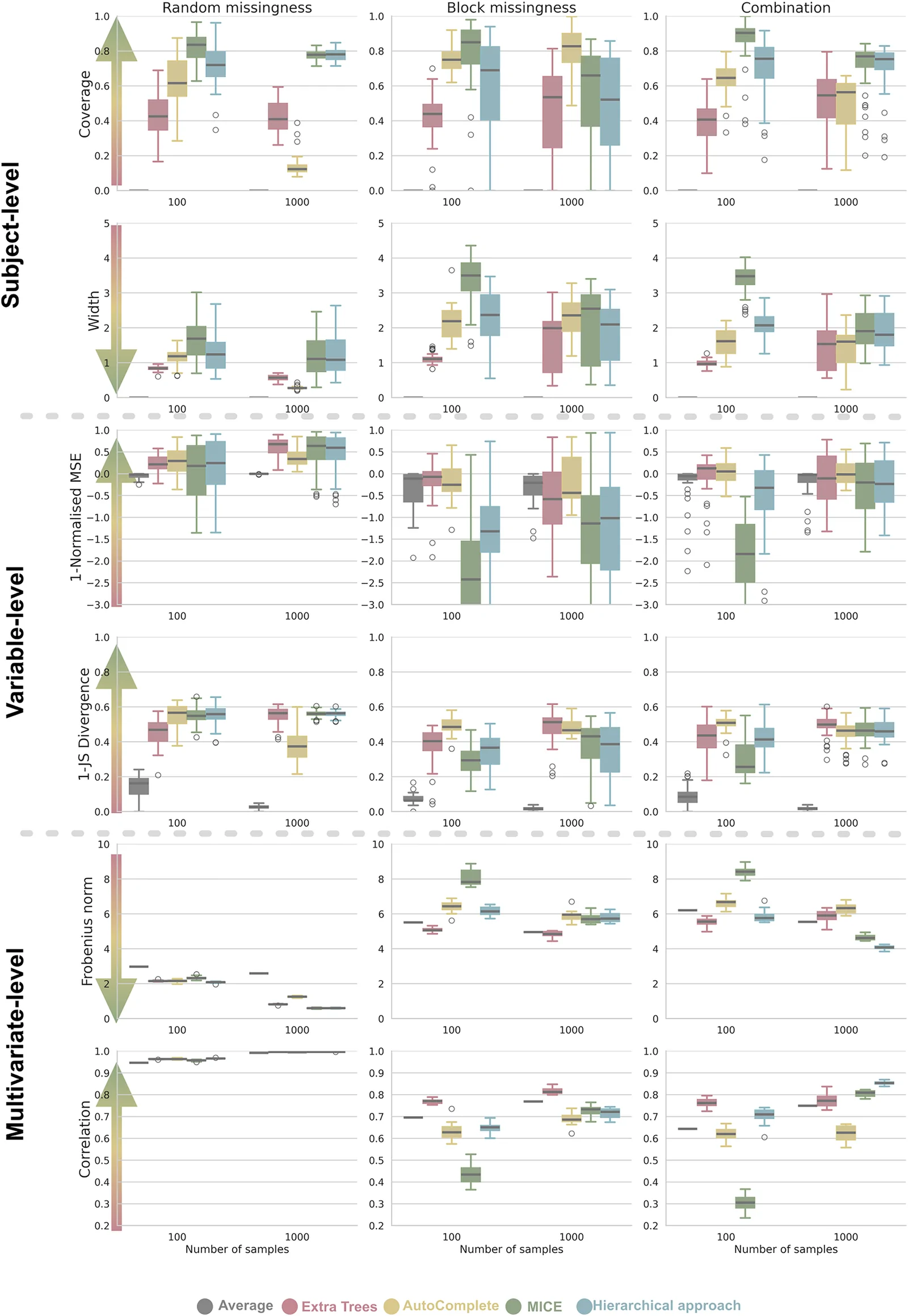
Evaluation of the imputation methods on the simulated datasets. Each row represents a quality of imputation criterion, each column represents a different type of missingness. The results are shown for five imputation algorithms: Average imputation, Extra Trees^41^, AutoComplete^42^, Multivariate Imputation by Chained Equations (MICE)^43^, and hierarchical MICE^44^; and for two sample sizes (100, 1000). Metrics were computed exclusively on originally missing entries, so the number of evaluated variables varies by missingness type and site (e.g. block missingness restricts evaluation to only the variables masked at a given site). The metrics used are: coverage and width (one value per evaluated variable, averaged across subjects with missing data), 1-normalised mean squared error (MSE) and 1-Jensen-Shannon (JS) divergence (one value per evaluated variable per *m* = 10 imputations), and Frobenius norm and correlation (n=10 values per boxplot, one per imputation). Exact n and range of values are provided in the Supplementary Data (file boxplot_measures, sheet Fig5). Here, the amount of random noise missingness was set to 30%, while both batteries overlapped in 10 variables. The arrows on the left side point in the direction of the ideal reconstruction values.

Applying these metrics to our simulated datasets revealed substantial differences in performance across imputation methods (Fig. 4). Even under random missingness, the performance of most methods was suboptimal, and significantly deteriorated with the introduction of structured missingness (Fig. 4). In the random missingness condition (first column of Fig. 4), MICE-based algorithms outperformed other methods at the subject level, achieving coverage close to 0.8, although at the cost of wider uncertainty intervals compared to Extra Trees and AutoComplete. Notably, AutoComplete’s coverage consistently declined with larger sample sizes as a consequence of its deterministic architecture optimising for reconstruction accuracy rather than uncertainty calibration, converging to increasingly overconfident predictions as more data became available (an effect absent under block missingness, where structural gaps prevent the model from forming a stable latent representation). At the variable level, all methods outperformed simple mean imputation in terms of normalised mean squared error, though MICE-based methods showed notably high variability in quality, which decreased with larger sample sizes. In terms of distribution preservation (quantified by 1-JSD), all algorithms performed comparably, except for AutoComplete, which struggled with larger sample sizes, and mean imputation, which collapsed the original data distribution to a single value (i.e. a spike in the distribution). At the multivariate level, hierarchical MICE best preserved relationships across variables, outperforming other methods in both Frobenius norm and correlation-based metrics. Overall, under the random missingness assumption (for which these algorithms were originally designed), the most notable performance differences appeared at the subject level, with MICE-based methods, particularly hierarchical MICE, demonstrating the most consistent results across all evaluation levels.

Introducing blockwise missingness (second column of Fig. 4) led to a marked increase in uncertainty across all methods, as evident by the widened boxplots and whisker ranges. At the subject level, AutoComplete outperformed standard MICE at higher sample sizes, while hierarchical MICE showed a notable decline in performance. Extra Trees maintained its average performance but exhibited greater variability in imputed values. At the variable level, both standard and hierarchical MICE struggled with NMSE, performing significantly worse than in the random missingness scenario. However, Extra Trees and AutoComplete also performed poorly, even worse than mean imputation. Multivariate relationships were also affected, as indicated by increased Frobenius norm values. Standard MICE, in particular, failed to maintain above-average performance in smaller samples, whereas hierarchical MICE and Extra Trees better preserved multivariate structures. Finally, when blockwise and random missingness were combined, results fell between the two extremes, with overall performance largely determined by the relative proportions of each missingness type.

The additional scenarios presented in Supplementary figures show trends consistent with those described above. Regardless of the size of the overlap or the percentage of random missingness, the overall patterns remain stable. In general, a lower degree of missingness leads to better imputation performance when combined with block missingness. Furthermore, the size of variable overlap between datasets plays a crucial role in preserving multivariate relationships: Smaller overlaps result in greater deterioration of these relationships.

### Real-world data analysis

**Fig. 5:**
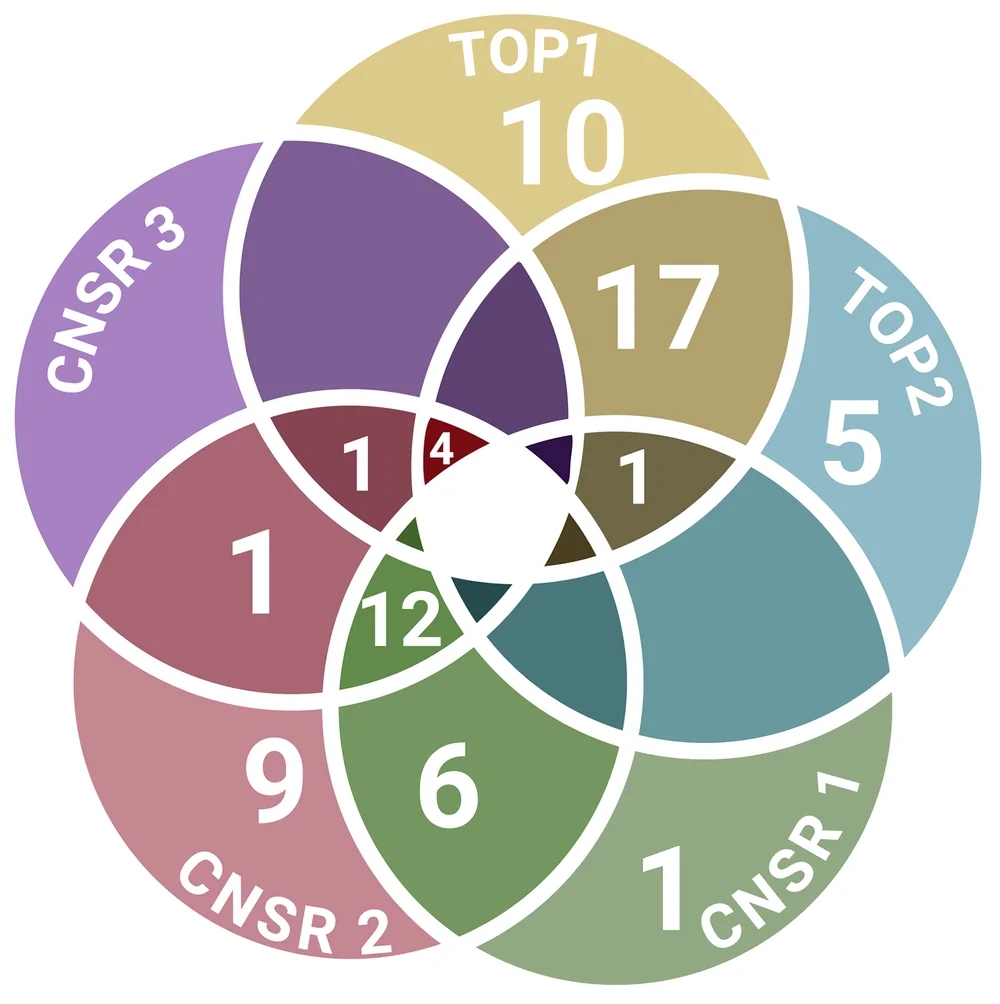
Overlap of conducted tests across the evaluated datasets. Each circle represents number of tests acquired in the respective dataset, the overlaps describe common variables between the datasets.

We subsequently applied this framework to two real-world scenarios of increasing complexity. First, we imputed cognitive data from the Norwegian Thematically Organised Psychosis (TOP) cohort^47,48^, comprising two overlapping test batteries (509 and 667 subjects, containing 37 unique variables Fig. 5) whose structured missingness arose from a mid-study transition between instruments. Second, we extended the analysis to include three additional cohorts from the Centre for Neuropsychiatric Schizophrenia Research (CNSR)^49–51^ (80, 119, and 100 subjects, containing 35 unique variables Fig. 5), introducing more pronounced site effects due to differences in language, testing personnel, and cohort composition. A full list of cognitive tests administered across all cohorts is provided in Supplementary Table. Because ground truth is unavailable for real-world missing data, we used a k-fold cross-validation approach^3^: the dataset was masked 20 times, each time randomly removing 5% of each variable’s observed values (in addition to already-missing values), allowing direct comparison between imputed and true values after reconstruction.

**Fig. 6:**
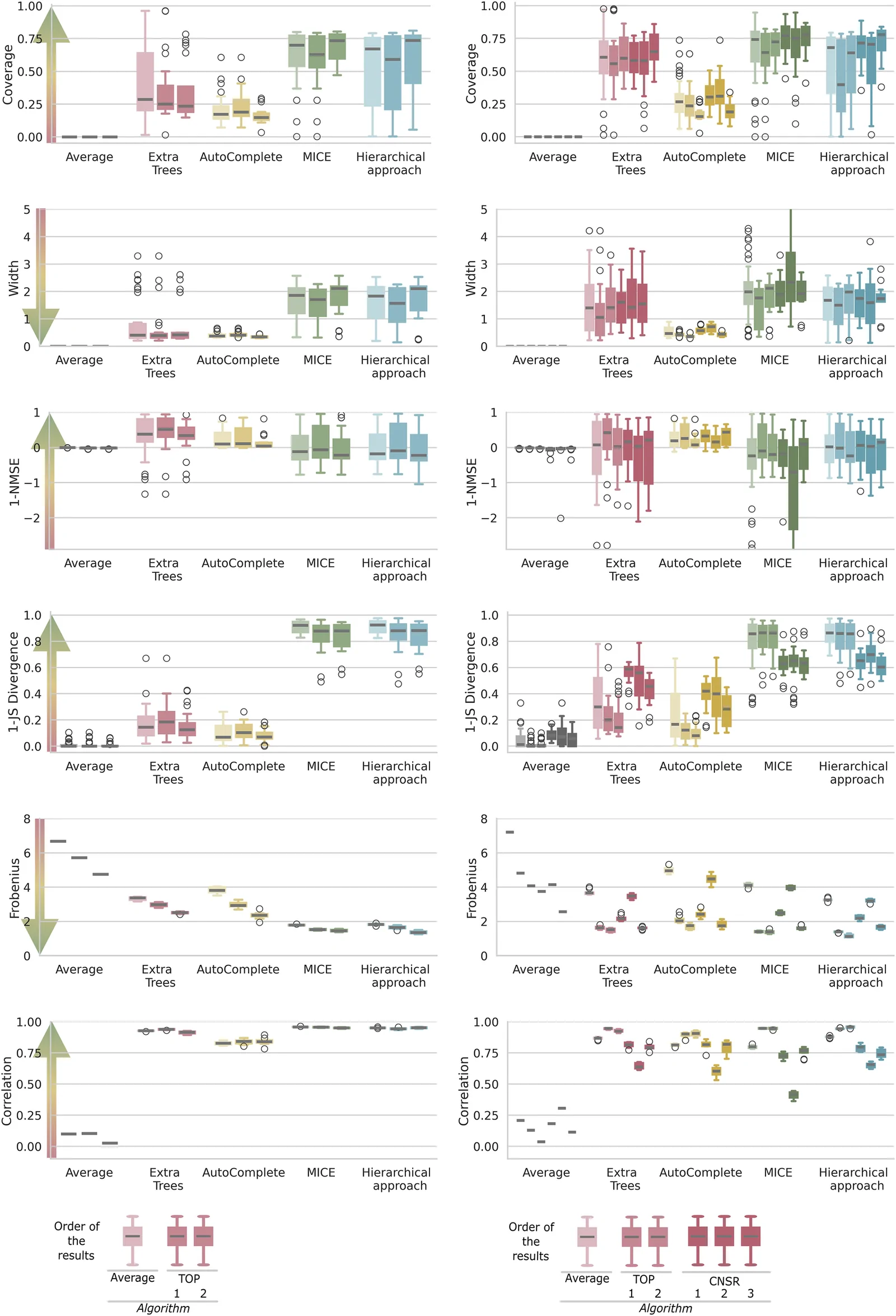
Evaluation of k-fol cross-validation on a real-world dataset. Each subplot presents boxplots for different imputation algorithms, colour-coded for distinction. Metrics were computed exclusively on originally missing entries, so the number of evaluated variables varies by site and by whether the pooled or single-site analysis is shown. The metrics used are: coverage and width (one value per evaluated variable, averaged across subjects with missing data), 1-normalised mean squared error (MSE) and 1-Jensen-Shannon (JS) divergence (one value per evaluated variable per *m* = 10 imputations), and Frobenius norm and correlation (*n* = 10 values per boxplot, one per imputation). Exact n and range of values are provided in the Supplementary Data (file boxplot_measures, sheet Fig6_TOP and Fig6_TOP_CNSR). The presented imputation algorithms are: Average imputation, Extra Trees^41^, AutoComplete^42^, Multivariate Imputation by Chained Equations (MICE)^43^, and hierarchical MICE^44^. Within each algorithm, the first (lighter-coloured) boxplot represents the quality measure computed on the entire dataset, while subsequent boxplots correspond to individual sites. The left panel shows results for the analysis using only the two cognitive batteries of the Thematically Organised Psychosis (TOP) cohort^47,48^, while the right panel extends the analysis to include three additional datasets from the Centre for Neuropsychiatric Schizophrenia Research (CNSR)^49 – 51^ (shown in darker colours). Arrows on the left indicate the direction of ideal reconstruction values.

Overall, the imputation results aligned closely with our simulation findings (left column of Fig. 6). Subject-specific metrics, such as coverage and interval width, were dominated by MICE-based approaches, whereas Extra Trees and AutoComplete produced imputed values with a much narrower range, failing to capture the true values. Consequently, these methods outperformed MICE-based approaches in the normalised mean squared error metric. The most notable deviation from our simulations was the extent to which MICE-based methods preserved the original data distribution. This advantage was further reflected in the preservation of multivariate relationships, particularly as measured by the Frobenius norm. Furthermore, as data from additional sites were integrated (right column of Fig. 6), imputation quality for the original two batteries did not significantly change. This resilience is encouraging, as it demonstrates that the imputation for the original datasets was not negatively affected by the new sources of variance introduced by the additional data. However, these evaluations only captured how well the algorithms reconstructed values that were originally observed without providing insight into the plausibility of imputations for values that had been missing from the outset.

**Fig. 7:**
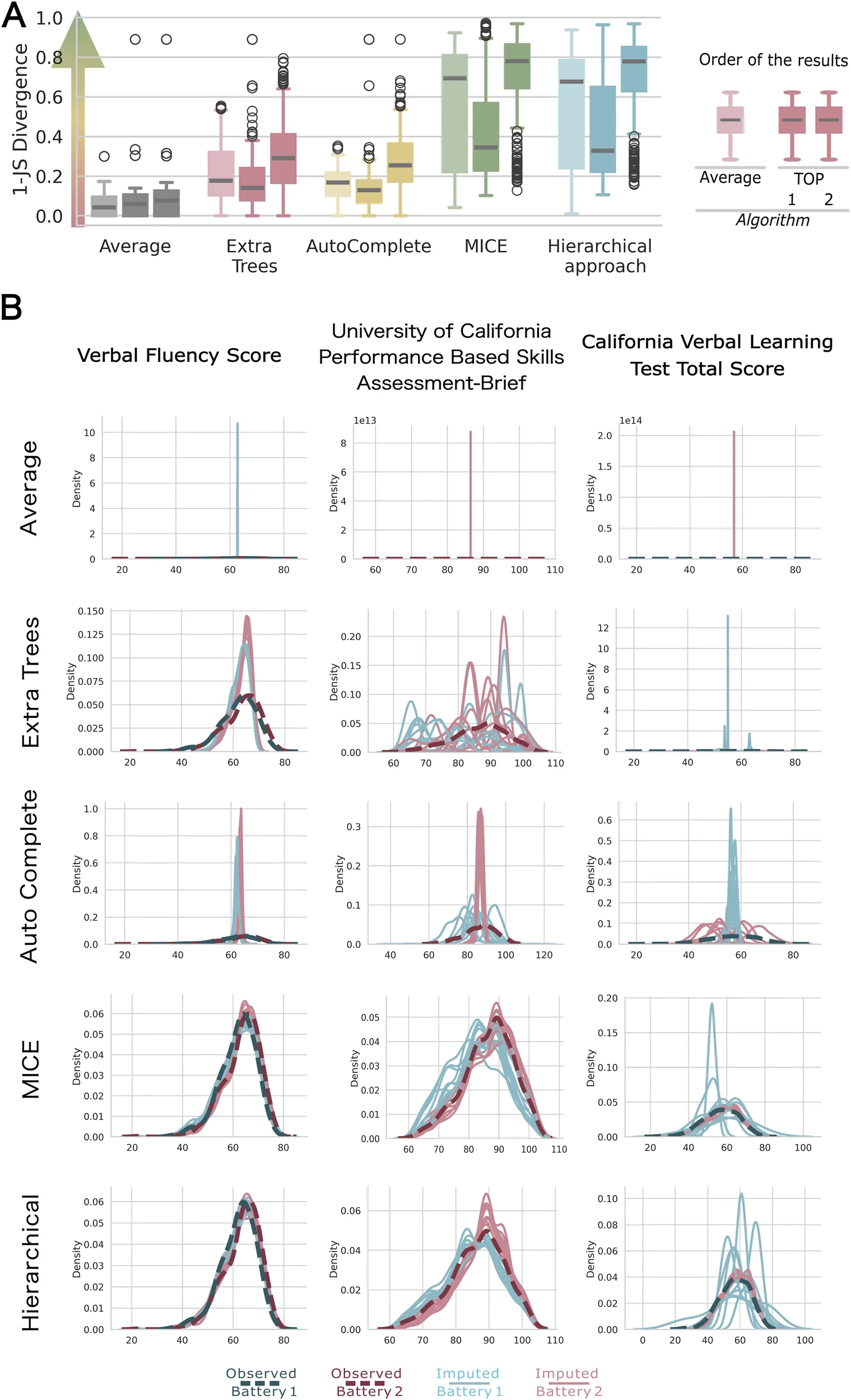
Validation on a real-world dataset. **A** Comparison of the imputed variable distributions with the original distributions in the Thematically Organised Psychosis (TOP) cohort^47,48^, quantified by 1-Jensen-Shannon (JS) divergence. Metrics were computed exclusively on originally missing entries, so the number of evaluated variables varies by site (370 pooled across both sites, 330 for TOP1, and 220 for TOP2); exact n and range of values are provided in the Supplementary Data (file boxplot_measures, sheet Fig7). The presented imputation algorithms are: Average imputation, Extra Trees^41^, AutoComplete^42^, Multivariate Imputation by Chained Equations (MICE)^43^, and hierarchical MICE^44^. **B** Examples of imputation approaches (rows) for cognitive variables (columns) across the two batteries of the TOP dataset-dark dashed lines represent the distribution of the original data, while thin lines indicate the distribution of the imputed data. In the first column, the variable was part of both TOP batteries, while in the second and third columns, the variable was only measured at the red and blue sites, respectively.

To assess the plausibility of imputations for genuinely (structurally) missing values, for which no ground truth exists, we additionally compared the distribution of imputed values against observed values at each site using Jensen-Shannon divergence. As shown in Fig. 7B, methods differed substantially in their ability to recover plausible distributions, particularly for variables measured at only one site. Specifically, AutoComplete produced overly narrow distributions incompatible with the original data, while Extra Trees showed highly variable distributions at the unmeasured site.

Both the standard MICE and the Hierarchical method outperformed the alternative approaches (Fig. 7). Fig. 7B clearly illustrates the shortcomings of individual approaches. The first column of density plots shows that most methods reconstructed the variable well when measured at both sites. However, in the other columns, where only one site measured the variable, Extra Trees performed poorly, with highly variable distributions fitted at the unmeasured site. AutoComplete, in contrast, adopted a narrow normal distribution to optimise absolute error, while MICE and the hierarchical method performed well, reflecting the distribution of the measured site. These figures show that despite an apparent equivalence in the summary statistics, the imputations produced by the hierarchical MICE are clearly superior to the standard implementation, showing much smoother distributions that have a better correspondence to the true distribution for the unmeasured site.

## Discussion

In this article, we have introduced a generic framework for handling structured patterns of missingness in biomedical datasets. Whilst our approach is widely applicable, it is particularly suited to modern ML and AI models that are trained on large scale cohorts, require complete data and focus on making out of sample predictions. We introduced a hierarchical simulation based framework to achieve these goals and validated this approach extensively using simulated and real data, showing good performance relative to a number of baselines.

Our results indicate that some approaches, like donor-based matching in MICE, are by design better suited to structured missingness than others, such as deep learning models or decision forests. While Extra Trees and AutoComplete performed well in previous benchmarks^3,42^, their reliance on numerical precision as the primary objective limited their effectiveness in the presence of structured missingness. Extra Trees, for instance, produced highly variable imputations when site effects were present, failing to capture the underlying data structure (Fig. 7). AutoComplete, by contrast, defaulted to narrow normal distributions, leading to overly confident predictions that did not reflect true variability. However, although these models are not ideally suited in their current form, future adaptations could improve their capabilities, for instance, by prioritising Kullback-Leibler divergence as an optimisation criterion in autoencoders, or site-aware regularisation in Extra Trees. However, progress in improving imputation methods will require moving beyond conventional accuracy metrics like mean squared error and considering evaluation strategies that capture structured missingness effects.

Our findings further highlight the critical role of preserving distributional properties and multivariate relationships when using imputation methods under structured missingness. Indeed, methods that maintained these properties, particularly the hierarchical MICE approach, demonstrated the preservation of both, thereby improving the robustness of the potential downstream analyses. As shown in Fig. 7, the hierarchical MICE method consistently produced imputations that better reflected the distribution of the measured site, even when entire variables were missing from a given location. These results underscore the importance of imputation strategies that do not optimise solely for a numerical error measure such as mean squared error. Our analysis of data from real-world cognitive batteries further underscores both the potential and limitations of imputation in addressing structured missingness. When site effects were minimal due to consistent data collection protocols, we were able to isolate the significant impact of structured missingness caused by the replacement of test batteries. In this setting, the hierarchical approach demonstrated its strength in preserving distributional and multivariate properties. As we expanded the analysis by incorporating additional datasets with more pronounced site effects, we observed a slight adjustment in performance for the initial datasets (Fig. 6). However, overall performance remained stable or even improved for most imputation methods, including the best-performing hierarchical approach.

Furthermore, our results highlight that conventional evaluation metrics, such as numerical precision, can be misleading under structured missingness. While all metrics reflected the impact of structured missingness, those focused on numerical precision, such as coverage, interval width, and normalised mean squared error, were disproportionately sensitive to site effects. In particular, when systematic shifts in means occurred across datasets, these methods failed to capture the true underlying distribution. This resulted in artificially low error measures even when the imputed values did not match the original structure. For example, if a site effect causes an offset in a variable, an imputation algorithm might correctly reconstruct individual values within one site while shifting them toward the mean of another. In such cases, 1-NMSE would be negative, and coverage would be low, even though the algorithm successfully preserved the general distribution of the variable. This example underscores a fundamental limitation of conventional error metrics: when structured missingness is present, optimising for numerical accuracy alone can degrade the integrity of the dataset. Indeed, as seen in our simulations, mean imputation will *by definition* achieve a lower normalised mean-square error while severely distorting the original distribution (Fig. 4).

The issue of misleading evaluation metrics becomes more pertinent with the increasing prevalence of multi-centre studies and data-sharing initiatives. As structured missingness arises across biomedical research due to site-specific assessment protocols, it is likely more widespread than previously recognised, and if unaddressed, may lead to systematic biases in multi-site datasets. This risk is particularly pronounced in precision medicine, where biased imputations can influence treatment recommendations. For example, in pharmacogenomics, systematic errors in imputing missing genotype data can lead to incorrect dosage recommendations, potentially compromising patient safety^52,53^. Furthermore, as demonstrated in our real-world example, it hinders the use of cognitive data across populations, and, when combined with neuroimaging measures, can obscure relationships between brain structure (or function) and behavioural outcomes. Multi-omics studies also face these challenges, as differences in platforms or batch effects systematically introduce missing data within or across sites. Consequently, these findings should be considered a starting point for further research into structured missingness and appropriate methods for mitigating its effects across various disciplines. A particularly challenging scenario arises when no variables are common across all sites, resulting in only transitive relationships across sites with no direct overlap. This scenario is becoming increasingly common as we move towards an era of large pooled datasets, compounding uncertainty in ways that standard evaluation metrics cannot fully capture, and raising the question of when imputation-based pooling remains defensible at all.

Our simulations offer a partial, data-driven answer to this question. As the overlap between sites shrank, the preservation of multivariate relationships deteriorated substantially across all methods (Supplementary Figs. S1–S9), reflecting a fundamental identifiability constraint rather than a shortcoming of any particular algorithm. This constraint is most severe when two datasets share no measured variables at all and no other dataset bridges the gap: in this fully disconnected case, there is no empirical path by which the joint distribution of the missing block can be anchored, and any imputation is necessarily determined entirely by prior modelling assumptions (e.g. multivariate normality, correlation stability across sites) rather than by information in the data. Our five-dataset real-world analysis illustrates a less severe, but still challenging, variant of this problem: no single variable was measured across all five cohorts, yet substantial pairwise overlap between subsets of datasets allowed relationships to be estimated transitively, propagating information (and uncertainty) across sites indirectly rather than through direct shared measurement. Such transitive imputation can still be informative, but its reliability depends on the strength and consistency of the intermediate links, and uncertainty compounds with each additional inferential step.

When datasets are genuinely disconnected (share no overlapping variables) imputation should not be used to manufacture inference it cannot support. In such cases, alternatives that avoid generating unobserved values altogether are preferable, such as analysing datasets separately and combining results via meta-analytic or federated approaches. Alternatively, collecting additional shared measures to re-establish an empirical link between datasets remains the most direct solution where feasible. More generally, we recommend imputation to be treated as appropriate only when sufficient shared structure exists to empirically constrain the missing values, and treated with substantial caution, including explicit sensitivity analyses, as that structure weakens.

**Fig. 8:**
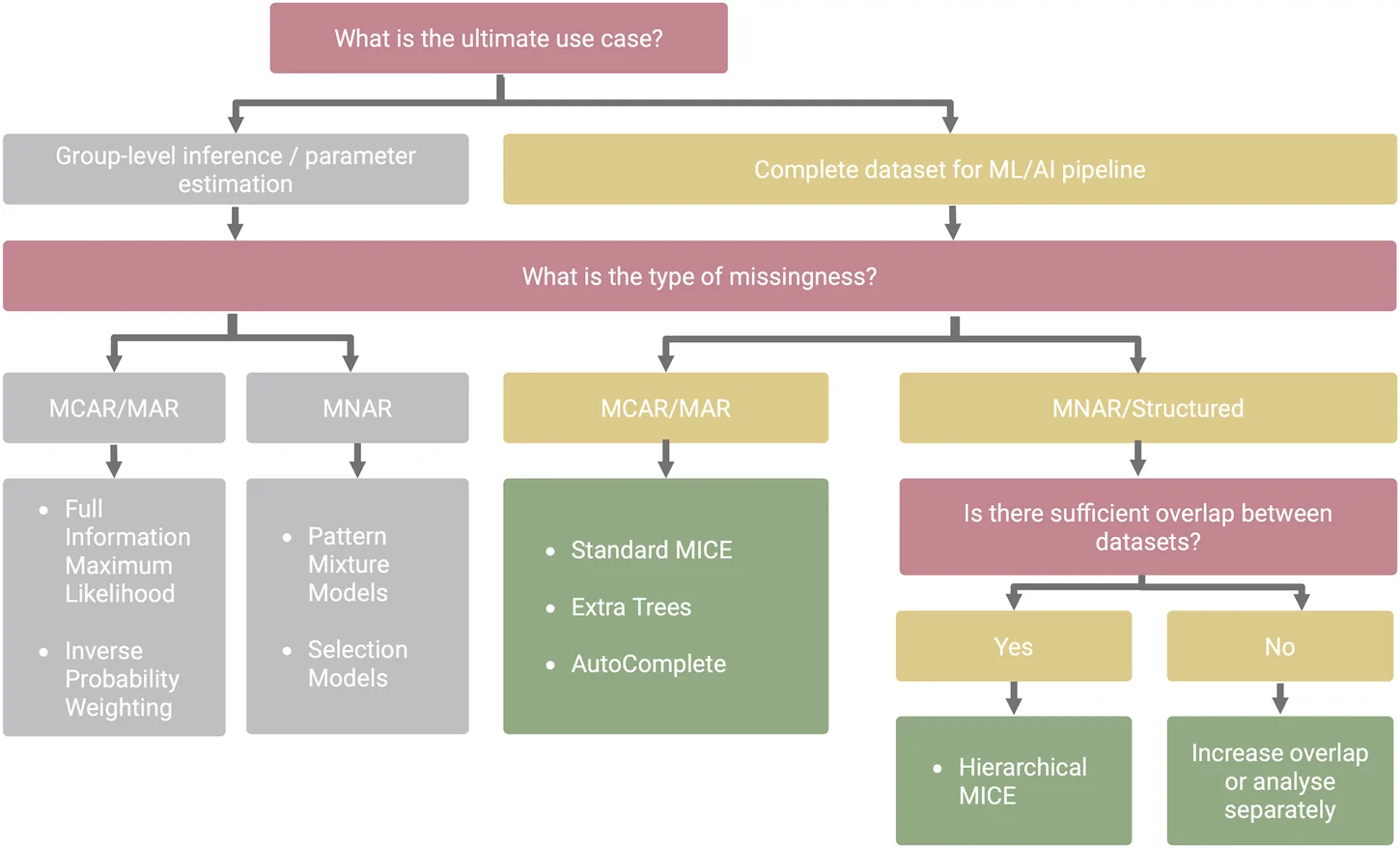
An overview of methods for handling missing data in biomedical research. Decision framework for selecting an appropriate missing data strategy based on the analytical goal and the type of missingness present. For group-level inference, full information maximum likelihood^9^ and inverse probability weighting^10^ are appropriate under missing completely at random (MCAR) or missing at random (MAR), while pattern mixture models^8^ and selection models^11^ address missing not at random (MNAR) scenarios. As group-level inference is outside the scope of this work, these branches are shown in grey. When the goal is to produce a complete dataset for downstream machine learning or AI applications, the choice of imputation strategy depends on the nature of missingness. Standard imputation methods suffice under MCAR/MAR, while structured missingness requires methods that explicitly account for site-specific variability. When sufficient overlap exists between datasets, hierarchical Multivariate Imputation by Chained Equations (MICE) is recommended^44^; when overlap is minimal, collecting additional overlapping variables or analysing datasets separately is advised. Created in BioRender. Rehak Buckova, B. (2026) https://BioRender.com/jgsuq1u.

More broadly, addressing missing data in biomedical research requires selecting an appropriate strategy based on both the analytical goal and the nature of the missingness present (Fig. 8). In this paper, we focused on producing complete datasets in order to make them compatible as preprocessing steps for ML/AI pipelines, which require complete feature matrices for all subjects, making imputation a necessary prerequisite. However, we do not claim that the approach we propose here is optimal in all cases. Where this constraint does not apply, other established methods offer a more principled solution: pattern mixture models^8^, full information maximum likelihood^9^, selection models^11^, and others model the data and missingness mechanism jointly, effectively bypassing the need to reconstruct missing values. We would thus recommend their use whenever the downstream analysis can accommodate them, reserving imputation for settings where the modelling approach itself demands a complete matrix.

Even where imputation is the appropriate tool, its performance depends on how well the missingness mechanism has been characterised. While our approach to simulating block missingness effectively captures a subset of real-world missingness patterns, it does not encompass the full complexity of structured missingness encountered in practice, such as batch effects, skip patterns, multimodal linkage and others outlined in Mitra et al.^7,24^. A deeper understanding of structured missingness will enable better integration of latent factors, variable dependencies, and dynamic missingness mechanisms, ultimately improving imputation strategies, as well as evaluation criteria^54,55^. However, even with improved theoretical understanding, bridging the gap between theory and the practical implementation of imputation methods across heterogeneous datasets remains a challenge^56^. If missingness patterns reflect demographic, socioeconomic, or systemic factors, employing imputation methods that ignore these disparities risks reinforcing biases, skewing analyses, and ultimately exacerbating health inequities^57^. Moreover, if datasets differ in their demographic makeup, where one dataset predominantly includes older adults while another focuses on adolescents, imputation models may not be able to generalise effectively, resulting in biased predictions and potentially flawed inferences^58^. Addressing these challenges requires thoughtful preprocessing and model design to mitigate risks of bias.

While imputation is the initial step of the data-analytical pipeline that recovers missing values in a form that preserves site-specific variability, meaningful analysis across multiple datasets requires additional steps to account for systematic site effects. Harmonisation enables meaningful comparisons across sites by transforming data into a common space. In many multi-site studies, adjusting for site effects is sufficient for group-level comparisons^1–3^. However, these transformations inevitably introduce uncertainty and reshape the dataset in ways that traditional modelling approaches may not fully accommodate. Imputation itself generates multiple plausible values per missing data point, whether it is in probabilistic approaches, where posterior distributions capture inherent uncertainty, or more deterministic approaches, where the algorithmic uncertainty can be estimated through multiple random initialisations. However, most ML algorithms are not designed to handle multiple versions of the imputed dataset, limiting the reliability of downstream analyses. Addressing this challenge requires methods that explicitly account for uncertainty and site variability.

**Fig. 9:**
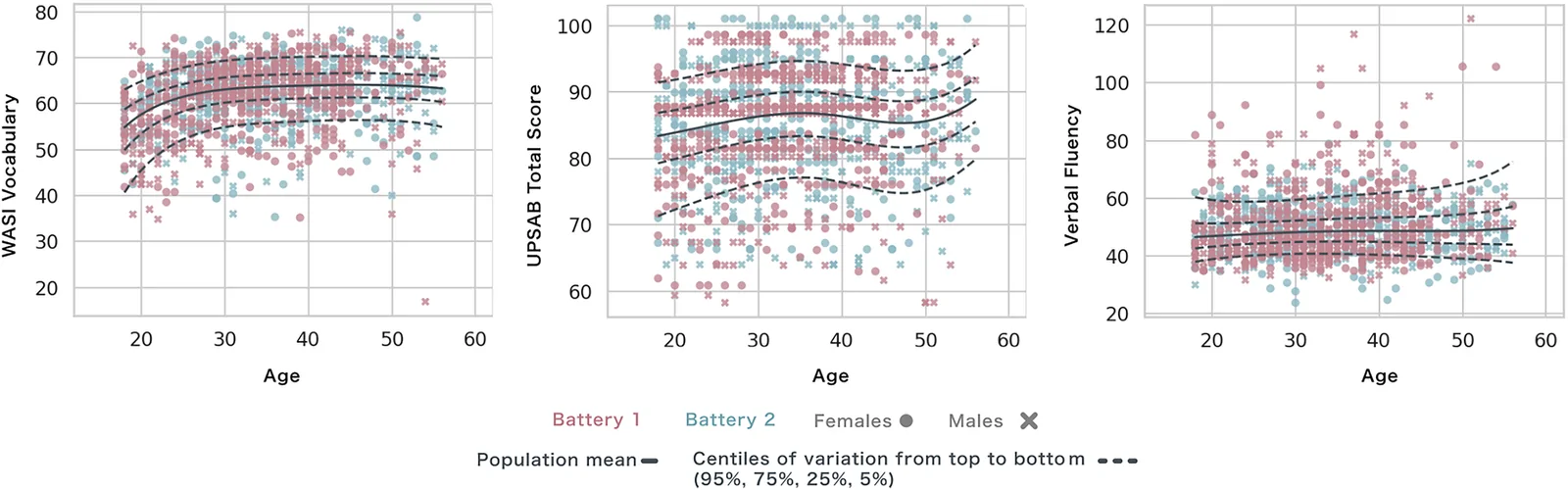
Suggested use with normative models. An example of normative models trained on the two imputed batteries of the Thematically Organised Psychosis cohort^47,48^ using the hierarchical method, depicted the variables shown in Fig. 7.

Normative modelling^59,60^ is particularly well-suited for this purpose. Rather than focusing on reconstructing absolute values, normative modelling emphasises preserving the underlying data distributions and multivariate relationships, making it robust to the uncertainties introduced by imputation and harmonisation. By constructing a universal population-wide model that can be adjusted for individual sites, normative modelling leverages the increased sample size while accounting for site-specific effects (Fig. 9). This approach is particularly advantageous when precise individual imputations are unreliable; instead, group-level imputations can recover the overall data structure, and predictions at the individual level are made only when data are actually observed. In doing so, normative modelling not only preserves biologically meaningful variability and risk indicators^4,61^, but also avoids the pitfalls of forcing uniformity across heterogeneous datasets.

In summary, our findings highlight the varied impact of structured missingness on the performance of imputation methods. We show that varied evaluation metrics are needed to evaluate the effects of structured missingness. Further, we highlight that a narrow focus on numerical precision alone risks obscuring underlying biases and site-specific variability, limiting the reliability of downstream analyses. By adopting approaches that preserve distributional integrity and multivariate relationships, we can better leverage heterogeneous, multi-site datasets to extract meaningful insights. This strategy enhances both subject-level inference and population-level modelling, as demonstrated by methods such as normative modelling. However, fully addressing the challenges posed by structured missingness requires the continued development of integrated imputation-harmonisation pipelines, improved fairness-aware validation frameworks, and adaptive modelling approaches capable of handling residual uncertainty. Ensuring robust and equitable imputation will be essential for advancing precision medicine and other areas of biomedical science.

## Methods

### Simulation study

Our simulation study comprised four distinct stages (Fig. 3). First, we synthesised complete datasets, thereby establishing a ground truth against which imputation accuracy could be assessed, a benchmark not generally available in observational studies where true missing values remain unknown. Second, we introduced different patterns of missingness into the dataset, thereby creating an amputated version on which the imputation algorithms would be tested. We incorporated both random and structured missingness patterns to capture a range of real-world scenarios. Next, we applied the imputation algorithms to reconstruct the missing data. To account for the inherent uncertainty of these algorithms, each is initialised 10 times using different random seeds, except for the MICE-based algorithms, which by default sample from matched donors for each variable without needing re-initialisation. Finally, we evaluated the imputation results using a comprehensive set of criteria. A detailed description of the process is outlined below.

### Dataset simulation

1. *Covariance structure*: a covariance matrix (Σ) and the mean vector (**μ**) were derived from a real dataset of cognition, ensuring realistic correlations between variables. This approach preserves the transitivity of the correlation matrix while adequately reflecting real-world scenarios. The covariance matrix was defined as:
  $${{\Sigma }}=\left[\begin{array}{cccc}{\sigma }_{{X}_{1},{X}_{1}}&{\sigma }_{{X}_{1},{X}_{2}}&\ldots \,&{\sigma }_{{X}_{1},{X}_{d}}\\ {\sigma }_{{X}_{2},{X}_{1}}&{\sigma }_{{X}_{2},{X}_{2}}&\ldots \,&{\sigma }_{{X}_{2},{X}_{d}}\\ \vdots &\vdots &\ddots &\vdots \\ {\sigma }_{{X}_{d},{X}_{1}}&{\sigma }_{{X}_{d},{X}_{2}}&\ldots \,&{\sigma }_{{X}_{d},{X}_{d}}\end{array}\right]$$
  where each entry ${\sigma }_{{X}_{i},{X}_{j}}$ represents the estimated covariance between variables *X*_*i*_ and *X*_*j*_.
2. *Data generation*: data were sampled from a multivariate normal distribution, with the mean vector and covariance matrix estimated from the cognitive dataset. To account for demographic effects, the data were conditioned on age (*X*_0_), which was generated uniformly between 18 and 60:
  $${X}_{0} \sim \,{\mbox{Uniform}}\,(18,60)$$
  The remaining variables were drawn from the conditional multivariate normal distribution given *X*_0_ = *x*_0_, which naturally captures the linear dependence of each variable on age:
  $${\mu }_{{{{\rm{cond}}}}}={\mu }_{{{{\bf{X}}}}}+{{{\Sigma }}}_{{{{\bf{X}}}},{X}_{0}}{{{\Sigma }}}_{{X}_{0},{X}_{0}}^{-1}({x}_{0}-{\mu }_{{X}_{0}})$$
  $${{{\Sigma }}}_{{{{\rm{cond}}}}}={{{\Sigma }}}_{{{{\bf{X}}}},{{{\bf{X}}}}}-{{{\Sigma }}}_{{{{\bf{X}}}},{X}_{0}}{{{\Sigma }}}_{{X}_{0},{X}_{0}}^{-1}{{{\Sigma }}}_{{X}_{0},{{{\bf{X}}}}}$$
  where ${{{\Sigma }}}_{{{{\bf{X}}}},{X}_{0}}$ represents the covariance between *X*_0_ and the remaining variables **X** = (*X*_1_, …, *X*_*d*_).
3. *Site-specific effects*: to introduce variability across datasets, each variable’s mean ${\mu }_{{X}_{i}}$ was independently shifted for each site by a randomly signed factor *δ*_*i*_:
  $${\mu }_{{X}_{i}}^{* }={\mu }_{{X}_{i}}+{\delta }_{i},\quad {\delta }_{i} \sim \,{\mbox{Uniform}}\,(-0.1{\mu }_{{X}_{i}},0.1{\mu }_{{X}_{i}}),$$
  where *δ*_*i*_ is sampled independently for each variable and each site, with the sign drawn randomly from {−1, +1}
4. *Sample sizes*: simulations were conducted with sample sizes of 100 and 1000, split evenly between the two datasets (50 and 500 per site, respectively). These sizes represent typical data scales in biomedical research, from exploratory studies to more extensive cohorts.

### Missing data mechanisms

We introduced three forms of missingness, each simulated independently and in combination to reflect common challenges in dataset integration. While this list is not exhaustive (see^24^ for a more comprehensive typology), it reflects several common scenarios encountered when merging heterogeneous datasets.

- *Random missingness*: for each variable *X*_*j*_, a predetermined percentage *p*_noise_ of values was set as missing at random. Missingness probability for each variable was applied uniformly across observations:
  $${P}_{{{{\rm{missing}}}}}({X}_{j})={p}_{{{{\rm{noise}}}}},\quad \,{\mbox{where}}{p}_{{{{\rm{noise}}}}}\in \{0.1,0.2,0.3\}$$
  This approach reflects the missing completely at random with no structure mechanism.
- *Blockwise missingness across datasets*: variables were randomly assigned to one of two datasets, *D*_1_ or *D*_2_, while maintaining a predefined overlap set of variables. The overlap size was set to 4, 10, or 20 variables to reflect low, medium, and high overlap scenarios. Variables outside the overlap set were equally divided between the two datasets, with each dataset receiving an exclusive subset of non-overlapping variables, which were deleted from the alternate dataset to create a blockwise missing structure:
  $${X}_{i}\in {D}_{1}\ \Rightarrow \ {X}_{i}\,\notin \,{D}_{2},\quad {X}_{i}\in {D}_{2}\ \Rightarrow \ {X}_{i}\,\notin \,{D}_{1}$$
  This approach simulates the multi-dataset contexts with partially overlapping variables.
- *Dependency missingness*: following^24^, dependencies in missingness were introduced between *two* selected columns. The pattern was implemented as follows:
  1. *Predictive variable standardisation*: for a predictor variable *X*_*i*_ shared across datasets, we standardised it by computing:
    $${A}_{{{{\rm{standardised}}}}}=\frac{{X}_{i}-{\mathbb{E}}[{X}_{i}]}{\sqrt{\,{\mbox{Var}}\,({X}_{i})}}$$
  2. *Probability of missingness*: missingness in a dependent variable *Y* was assigned based on the standardised predictor’s deviation, using:
    $${P}_{{{{\rm{missing}}}}}(Y)=\frac{\exp (\alpha \cdot | {A}_{{{{\rm{standardised}}}}}| )}{1+\exp (\alpha \cdot | {A}_{{{{\rm{standardised}}}}}| )}$$
    where *α* = 3 controls the likelihood of missingness based on the predictor’s distribution characteristics.
  3. *Conditional missingness in additional variables*: for a third variable *Z*, missingness was introduced conditionally on *Y*’s missing status:
    $${P}_{{{{\rm{missing}}}}}(Z| {\mbox{is missing}}\,(Y))=q$$
    with *q* = *missingness*, specifying a structured, conditional missingness pattern.

### Imputation methods

To recover incomplete datasets, we applied four state-of-the-art imputation algorithms (Table 1), each representing a distinct computational approach to imputing missing data.

Extremely Randomised Trees (Extra Trees) is a tree-based ensemble method for classification and regression that introduces additional randomness during tree construction by randomising both the feature selection and split threshold choice^41^. Unlike Random Forests, which select the best split from a random subset of features, Extra Trees chooses the feature and the cut-point completely at random, promoting greater diversity among the trees in the ensemble and improving robustness in some cases^41,62^. Furthermore, unlike in the Random Forest case, each decision tree in the ensemble is built on the entire dataset (without bootstrapping). We have chosen this method, as it has been previously shown to adapt well to nonlinearities in datasets, often a desirable property for imputation in complex datasets^3,63^. We took advantage of the Python implementation of random forests, combined with the Iterative Imputer implementation for imputation.

AutoComplete is a recently introduced imputation algorithm aimed at handling extensive phenotypic missingness in large-scale biobank datasets^42^. Using a copy-masking mechanism within an autoencoder architecture, it learns patterns within incomplete data to impute both binary and continuous phenotypes. Copy-masking allows the algorithm to incorporate partially observed data by selectively masking inputs, effectively increasing sample size suitable for training as well as improving imputation robustness. While AutoComplete demonstrated improved performance over traditional imputation methods, it has not yet been evaluated on datasets with block-structured missingness, leaving open questions about its effectiveness with structured missingness scenarios, a common feature in integrated biobank data.

The Fully Conditional Specification approach, commonly implemented as MICE^43^, is a widely adopted framework for multiple imputation. MICE operates iteratively over 20 inner cycles (maxit = 20), specifying a conditional model for each variable given all others, and then draws imputed values from the (approximate) posterior predictive distribution of these models, generating *m* = 10 multiple iterations per run. This inherent stochasticity, rather than imputing a single best prediction, introduces appropriate random variation, crucial for preserving data variance and reflecting imputation uncertainty. In this study, we utilised Predictive Mean Matching within the MICE framework, with a donor pool of *k* = 10 candidates. This algorithm first fits a temporary regression model using observed data to predict values for missing entries. It then identifies the k-closest donor cases from the observed data whose predicted values are closest to that of the missing entry. Finally, an actual observed value is randomly selected from one of these donors as the imputation, thereby ensuring that imputed values are plausible and drawn from the observed data distribution.

To account for multi-site variability, we implemented a hierarchical extension of the MICE^44^, explicitly modelling site-specific offsets for variables measured across multiple locations. This approach ensures that local differences are preserved while maintaining consistency across the dataset. Variables were first classified based on their site-specific missingness patterns. Those measured across multiple sites were flagged for hierarchical imputation, while fully observed variables remained unchanged. A *prediction matrix* was constructed using pairwise correlations, retaining only variable pairs with sufficient non-missing observations. Site-specific effects were incorporated by introducing an offset for multi-site variables, modelled as:

$${X}_{i,j}={\beta }_{0,j}+{\beta }_{1,j}{{\mbox{Site}}}_{i}+{\epsilon }_{i,j},\quad {\epsilon }_{i,j} \sim {{{\mathcal{N}}}}(0,{\sigma }_{j}^{2})$$

where *β*_1*j*_ captures site-level deviations. Single-site variables were imputed without this adjustment. Such a strategy balances local variability with overall coherence, refining imputation accuracy in multi-site studies.

Finally, we benchmarked all methods against simple average imputation, which replaces missing values with the variable mean. Although we do not recommend this approach, it is widely used in practice for its simplicity and computational efficiency, so we include it to illustrate its shortcomings and highlight the need for more advanced methods.

A notable difference among the tested algorithms is their ability to quantify uncertainty in the imputed values. MICE inherently captures uncertainty by generating *m* = 10 multiple imputations through sampling from the posterior predictive distribution, which is a statistically principled approach where variability across imputations reflects genuine uncertainty. In contrast, tree- and neural-network-based methods such as Extra Trees and AutoComplete lack an uncertainty estimate and were therefore run 10 times with distinct random seeds to approximate a distribution of imputed values.

### Simulation evaluation

We evaluated imputation quality at three levels, namely at the level of subjects, individual variables, and multivariate relationships (Fig. 3). All metrics were computed exclusively on originally missing entries.

At the subject level, we measured the *coverage*: the proportion of true values falling within the 95% percentile interval constructed across the *m* = 10 imputations for each subject; and *interval width*, normalised by the standard deviation of each variable, to guard against excessively broad uncertainty estimates. These measures are computed per variable and averaged across subjects with missing values, yielding one value per variable. We note that *m* = 10 imputations yield crude subject-level percentile intervals from a classical inference perspective, as the 95% interval is effectively determined by the minimum and maximum of 10 draws. However, coverage and interval width are used here as comparative measures of algorithmic uncertainty across methods rather than as calibrated subject-level predictions, for which purpose this is sufficient^64^. Well-calibrated subject-level uncertainty estimates for clinical applications require a larger number of imputations (e.g. 20–100 depending on the nature of missingness).

At the variable level, we use the mean squared error normalised by the variance of each variable to avoid the disproportionate influence of variables with larger numerical ranges. Specifically, we report 1-NMSE for easier interpretation (1 indicates perfect imputation, 0 performance equal to imputing with the variable’s mean, <0 indicates worse-than-average imputation).

We also use Jensen-Shannon divergence (Eq. (1)) to assess whether the original distribution of the data has been preserved. As JSD is bounded between 0 and 1, it is more suitable for comparing variables with different scales. *p* and *q* denote the probability distributions of the observed and imputed data for originally missing entries, respectively, and $m=\frac{1}{2}(p+q)$ is their average. For consistency, we report 1-JSD, where a value of 1 indicates perfect alignment between the original and imputed distributions, and a value of 0 indicates completely dissimilar distributions.

1 $$\,{{{\rm{JSD}}}}\,(p\parallel q)=\frac{D(p\parallel m)+D(q\parallel m)}{2}$$

Finally, the quality of imputation has been shown to significantly influence second-stage ML outcomes and algorithmic fairness^15,16^. However, the preservation of these complex relationships is often overlooked in imputation evaluations. Here, we use the Frobenius norm, calculated as the difference between the original and imputed correlation matrix. The second metric is the correlation of the vectorised upper triangles of the two matrices. Both metrics are computed per imputation and averaged across *m* = 10 imputations with the distributions shown as boxplots (Figs. 4 and 6). The Frobenius norm provides a summary measure of how well the overall correlations are preserved during imputation, while the ‘correlation of correlations’ captures whether the relationships between variables are retained or if systematic distortions emerge.

### Example data

As a practical example, we applied this framework to two real-world problems of increasing complexity.

First, we analysed cognitive battery data from the healthy subjects of the TOP cohort (Table 2)^47,48^. The dataset comprised two cognitive test batteries of 509 and 667 subjects, with 22 overlapping scores and a set of additional unique scores for each battery (11 and 4, respectively, Fig. 5). A full list of cognitive tests administered across all cohorts, along with descriptive statistics for each, is provided in Supplementary Table. This dataset offers a controlled setting where common sources of structured missingness, such as variations in language, personnel, and testing location, are minimised. Instead, missingness arose specifically from a transition between two cognitive test batteries (Battery 1, 2004–2012 and Battery 2, 2012–2023), which were designed to measure similar constructs but used different tests.

**Table 2:** Demographics of the samples stratified by datasets

	Site	Sex	Count	Age (min, max)
		All	509	34.6 (18, 56)
	1	Females	254	34.7 (18, 55)
TOP		Males	255	34.4 (18, 56)
		All	667	33.6 (18, 56)
	2	Females	306	33.3 (18, 56)
		Males	361	33.9 (18, 56)
		All	80	26.9 (18, 39)
	1	Females	25	26.9 (18, 38)
		Males	55	27 (20, 39)
	2	All	119	23.6 (18, 42)
CNSR		Females	61	22.1 (18, 34)
		Males	58	25.2 (19, 42)
	3	All	100	23.5 (18, 38)
		Females	56	22.3 (18, 33)
		Males	44	25 (19, 38)

In the second scenario, we incorporated three additional datasets collected at the CNSR in Denmark^49–51^. These datasets were acquired sequentially as part of various studies (Table 2), capturing mostly, but not entirely, overlapping variables (Fig. 5). Introducing additional datasets further challenged the imputation algorithms. First, site effects were expected to be more pronounced, as data collection occurred in a different language (implying a translated variant of the test), with different staff, and on a distinct cohort. At the same time, integrating additional datasets increased overall sample diversity, which could ultimately improve downstream analyses, as statistical and ML algorithms fundamentally rely on identifying and leveraging multivariate patterns in the data.

### Ethics

All clinical studies were conducted in accordance with the Helsinki declaration, and approval was obtained via the local ethical review authorities, specifically: The TOP dataset was approved by the Regional Committee for Medical Research Ethics and the Norwegian Data Protection Authority; studies CNSR 2 and CNSR 3 comply with the Committee on Biomedical Research Ethics (H-KF-01-78/97, H-D-2008-088, H-3-2013-149); CNSR 1 studies were approved by the Ethics Committees of Copenhagen and Frederiksberg/The Capital Region (KF01-305/99, H-KF-01-078/97).

### Example evaluation

To evaluate imputation performance against ground truth, we followed the k-fold cross-validation approach of Llera et al.^3^. Specifically, we masked the original merged dataset 20 times, randomly masking out 5% of each variable’s values (without repetition), on top of the values that were already missing. This ensured that, alongside imputing originally missing values, we also imputed a subset of values for which we had ground-truth data. After all iterations, we could fully reconstruct the original dataset, enabling a direct comparison between the imputed and true values. As in the simulation study, each algorithm was initialised 10 times per mask to assess variability in imputation outcomes.

To specifically address structured missingness (for which no ground truth exists by definition), we additionally employed Jensen-Shannon divergence to assess the plausibility of imputed values. For each variable, we compared the distribution of imputed values to that of the observed values at each site. For variables measured at both sites, an optimal imputation should produce distributions consistent with the site-specific observed data. For variables measured at only one site, the imputed distribution at the unobserved site should represent a plausible approximation informed by the available data from other sites. If no values were missing for a given variable, JSD was not estimated.

## Supplementary information

Supplementary information

Supplementary Data

## Data Availability

Data produced through simulations can be re-generated using the accompanying https://github.com/bbuckova/The-Art-of-Not-Knowing-Accommodating-Structured-Missingness-in-Biomedical-Research (GitHub repository), archived at Zenodo^40^. All data underlying the Figures (Figs. 1–8 in the main text and the Supplement) are available for download in the repository and as Supplementary_Data.zip. The repository contains: CSV files containing the per-variable, per-algorithm, per-seed values underlying all main and Supplementary Figures; boxplot_measures.xlsx: summary statistics (*n*, min, max) for each boxplot shown in Figs. 5–7, organised by sheet as described in the corresponding figure legends. Both files are also available for download via the https://github.com/bbuckova/The-Art-of-Not-Knowing-Accommodating-Structured-Missingness-in-Biomedical-Research (GitHub repository). As both cohorts contain identifiable clinical information, they are not publicly available via a data repository. Data from the Thematically Organised Psychosis (TOP) cohort^47,48^, and from the Centre for Neuropsychiatric Schizophrenia Research (CNSR) cohorts^49–51^ are stored on secure servers at their respective institutions and may be made available for collaborative re-use under a data-sharing agreement. Requests should be directed to Ole Andreassen (ole.andreassen@medisin.uio.no) and Bjørn Ebdrup (bjoern.ebdrup@regionh.dk), respectively. The request must include a research proposal describing the intended use; access is subject to execution of a data-sharing agreement compliant with (GDPR/relevant national regulation). Responses to data-sharing requests will be provided within 1 month.

## Code availability

Code for the simulation part of the study, together with requirements for its successful deployment, is available at https://github.com/bbuckova/The-Art-of-Not-Knowing-Accommodating-Structured-Missingness-in-Biomedical-Research (GitHub repository), archived at Zenodo^40^.
