# Supplementary material for: Structured missingness in biomedical research
Source: Commun AI Comput. 2026 Oct 9;1(1):19. doi: 10.1038/s44488-026-00025-9 (PMC13652732; doi:10.1038/s44488-026-00025-9)
Supplement: Supplementary file 1 [file 44488_2026_25_MOESM1_ESM.pdf]

Supplementary Information for: Structured Missingness in Biomedical Research

Barbora Rehák Bučková[1,2], Charlotte Fraza[1,2], Cecilie Koldbæk Lemvigh[3], Camilla Bärthel Flaaten[4], Linn Sofie Sæther[5], Giulia Cattarinussi[6], Karen Marie Sandø Ambrosen[3], Ole Andreas Andreassen[5,7], Lars Tjelta Westlye[7,8], Christian Beckmann[1,2], Bjørn Hylsebeck Ebdrup[3,9], Paola Dazzan[6,10], Torill Ueland[4,8], Robin Mitra[11], and Andre Marquand[1,2,*]

[1] *The Department of Medical Neuroscience of Radboud University Medical Centre, Nijmegen, Netherlands*

[2] *Donders Institute for Brain, Cognition and Behaviour, Radboud University Nijmegen, Nijmegen, Netherlands*

[3] *Center for Neuropsychiatric Schizophrenia Research and Center for Clinical Intervention and Neuropsychiatric Schizophrenia Research, Mental Health Centre Glostrup, Copenhagen University Hospital - Mental Health Services CPH, Copenhagen, Denmark*

[4] *Section for Clinical Psychosis Research, Division of Mental Health and Addiction, Oslo University Hospital, Oslo, Norway*

[5] *Centre for Precision Psychiatry, Division of Mental Health and Addiction, Institute of Clinical Medicine, University of Oslo, Oslo, Norway*

[6] *Department of Psychological Medicine, Institute of Psychiatry, Psychology and Neuroscience, King's College London, United Kingdom*

[7] *Section for Precision Psychiatry, Division of Mental Health and Addiction, Oslo University Hospital, and University of Oslo, Oslo, Norway*

[8] *Department of Psychology, University of Oslo, Oslo, Norway*

[9] *Department of Clinical Medicine, Faculty of Health and Medical Sciences, University of Copenhagen, Copenhagen, Denmark*

[10] *National Institute for Health and Care Research (NIHR) Maudsley Biomedical Research Centre at South London and Maudsley NHS Foundation Trust and King's College London, London, UK*

[11] *Department of Statistical Science, University College London, London, UK*

[*] *Corresponding author: Andre Marquand andre.marquand@donders.ru.nl*

Supplementary Figures

Here, we present additional results from the simulation study, specifically examining the effects of varying levels of added random noise, both in the context of random missingness and when combined with block and dependence missingness. The purpose of this extension is to assess the robustness of imputation algorithms under different noise conditions, simulating real-world variability in multi-site datasets.

We consider scenarios with overlapping variable sets of sizes 4, 10, and 20, introducing random noise to 10%, 30%, and 50% of values. This noise affects only the randomly missing and dependent missing values but does not impact block missingness, which remains at 100% for a given site by definition.

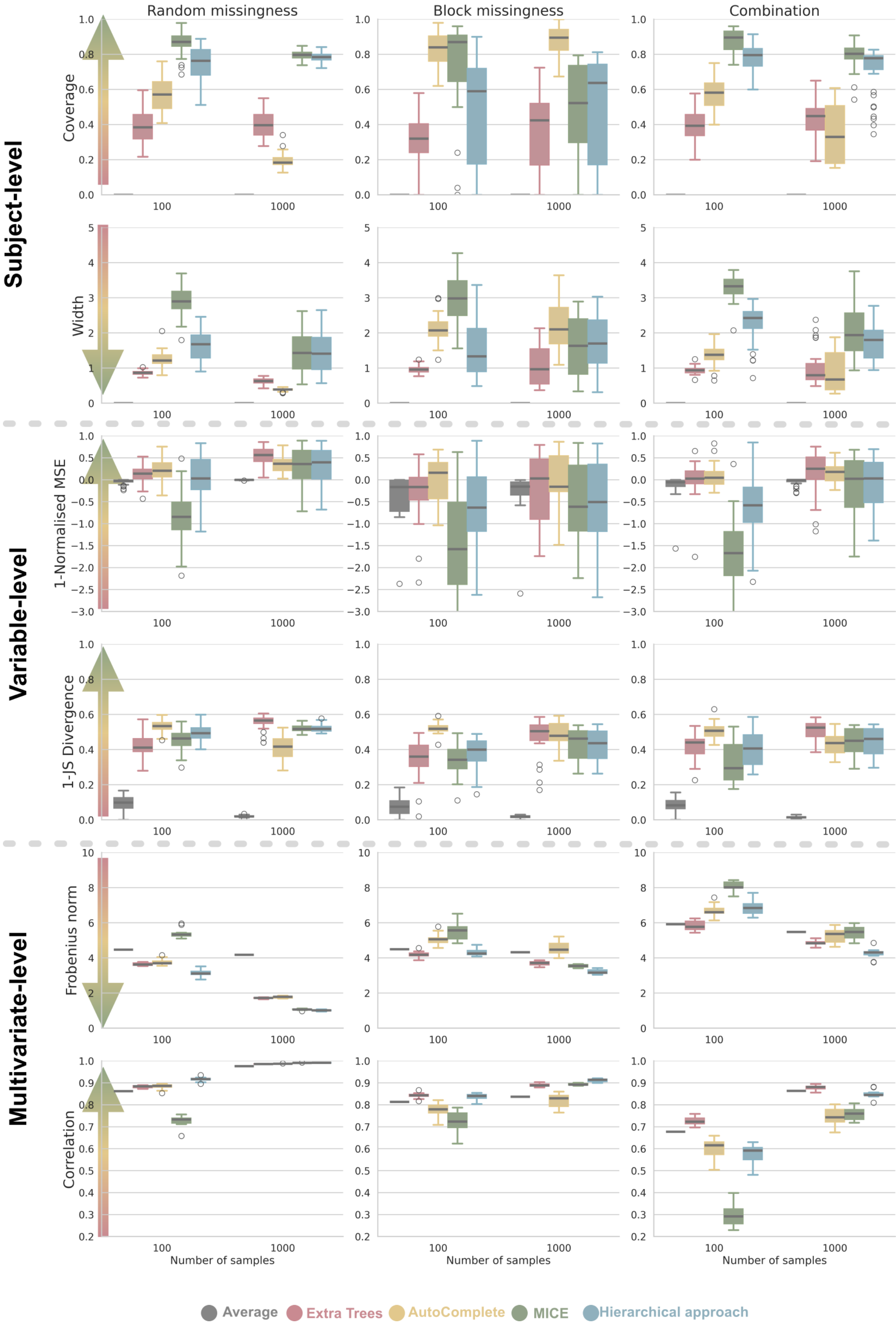

Figure S1: Evaluation of the imputation methods on the simulated datasets: Each row represents a quality of imputation criterion, and each column represents a different type of missingness. The results are shown for five imputation algorithms: Average imputation, Extra Trees, AutoComplete, MICE, and hierarchical MICE; and for two different dataset sizes (100, 1000). Here, the amount of random noise missingness was set to 50%, while both batteries overlapped in 20 variables. The arrows on the left side point in the direction of the ideal reconstruction values.

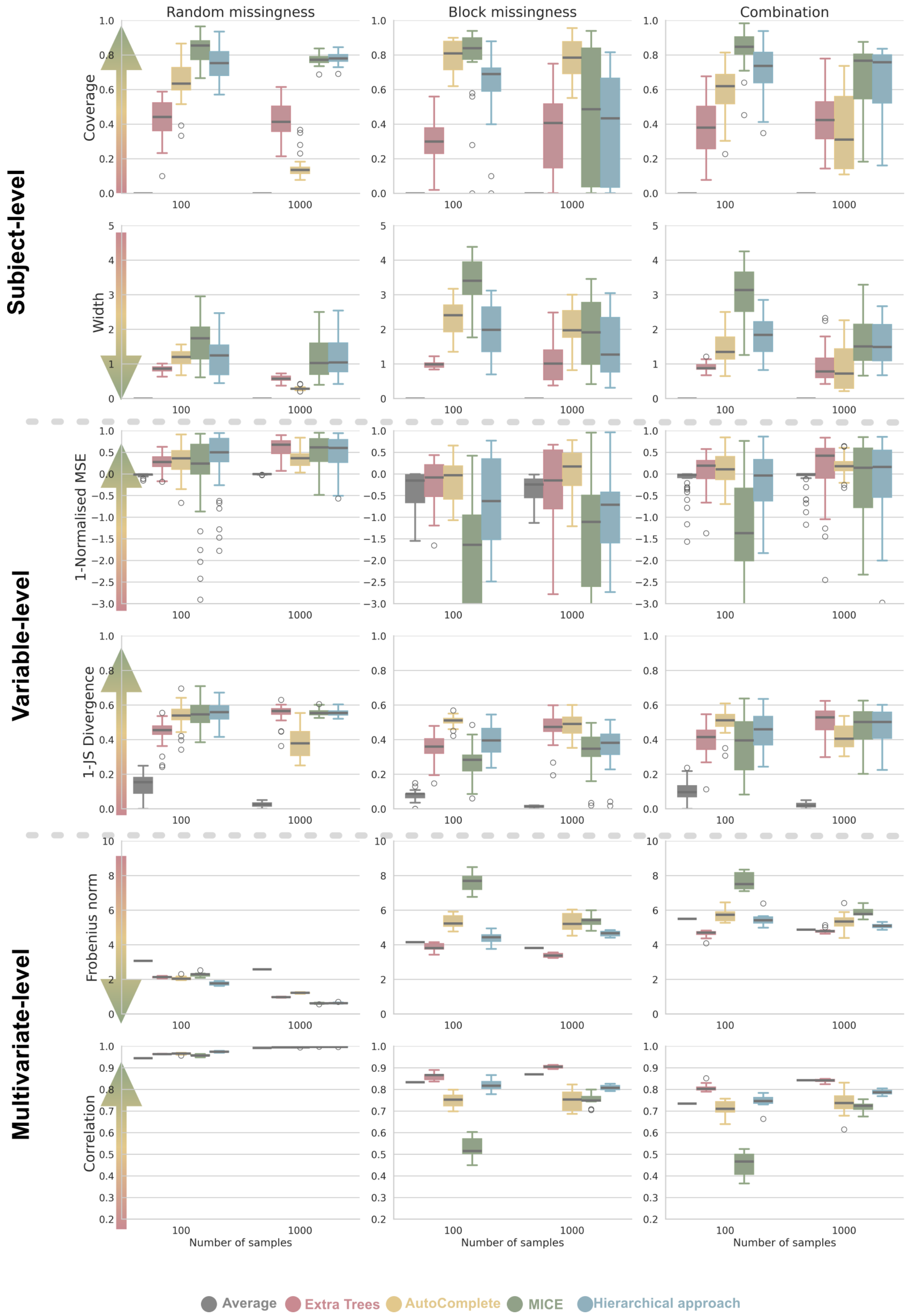

Figure S2: Evaluation of the imputation methods on the simulated datasets: Each row represents a quality of imputation criterion, and each column represents a different type of missingness. The results are shown for five imputation algorithms: Average imputation, Extra Trees, AutoComplete, MICE, and hierarchical MICE; and for two different dataset sizes (100, 1000). Here, the amount of random noise missingness was set to 30%, while both batteries overlapped in 20 variables. The arrows on the left side point in the direction of the ideal reconstruction values.

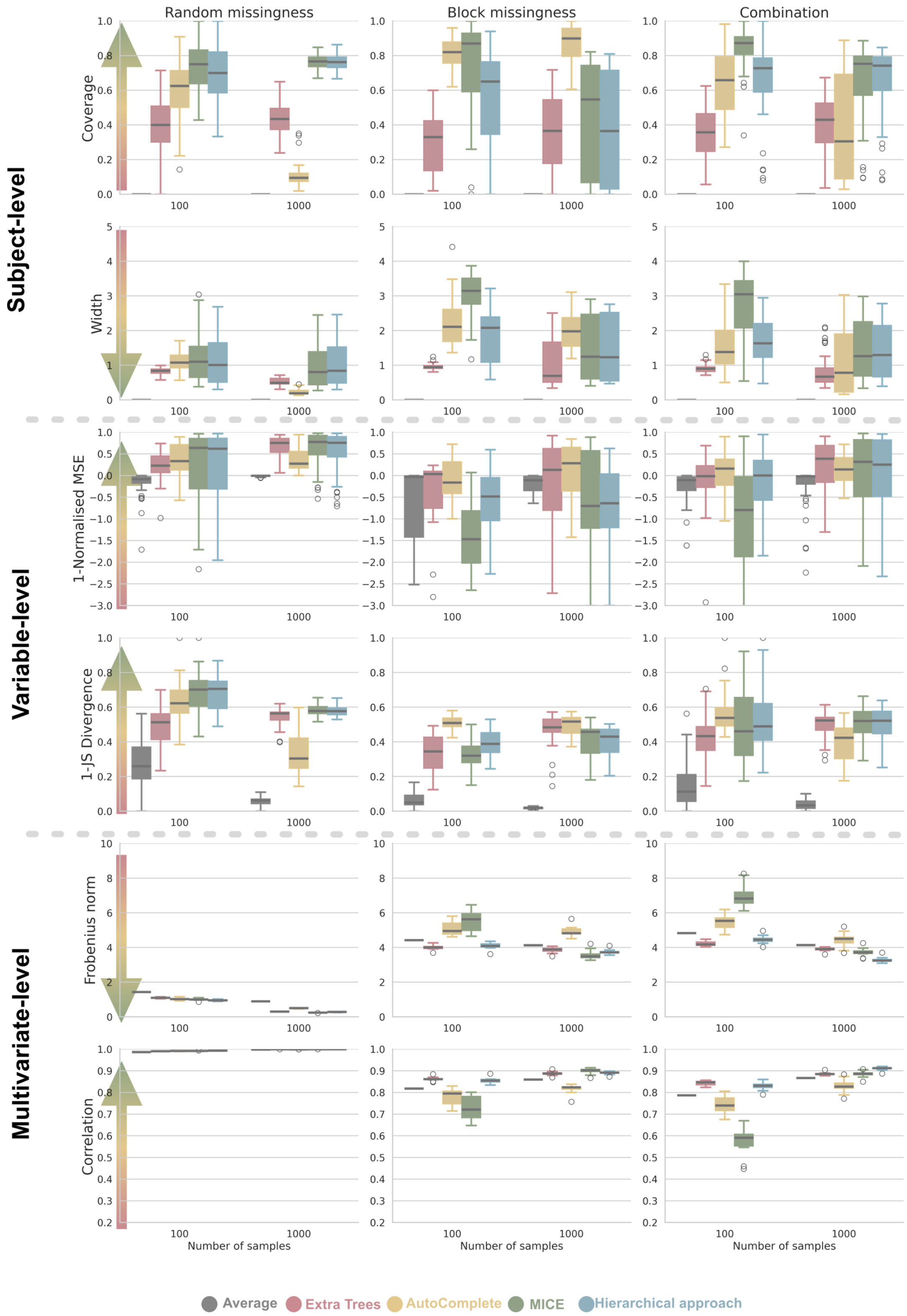

Figure S3: Evaluation of the imputation methods on the simulated datasets: Each row represents a quality of imputation criterion, and each column represents a different type of missingness. The results are shown for five imputation algorithms: Average imputation, Extra Trees, AutoComplete, MICE, and hierarchical MICE; and for two different dataset sizes (100, 1000). Here, the amount of random noise missingness was set to 10%, while both batteries overlapped in 20 variables. The arrows on the left side point in the direction of the ideal reconstruction values.

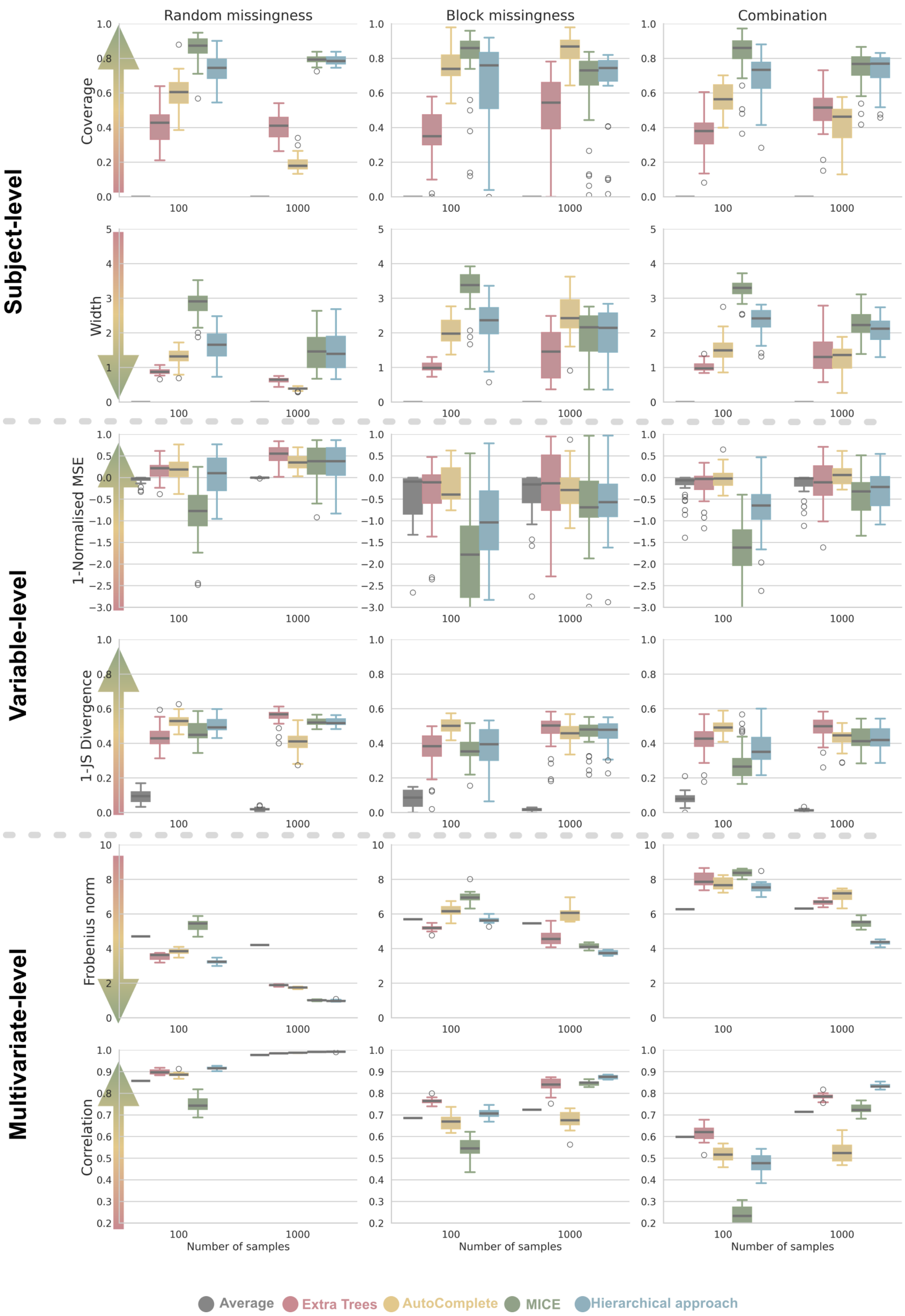

Figure S4: Evaluation of the imputation methods on the simulated datasets: Each row represents a quality of imputation criterion, and each column represents a different type of missingness. The results are shown for five imputation algorithms: Average imputation, Extra Trees, AutoComplete, MICE, and hierarchical MICE; and for two different dataset sizes (100, 1000). Here, the amount of random noise missingness was set to 50%, while both batteries overlapped in 10 variables. The arrows on the left side point in the direction of the ideal reconstruction values.

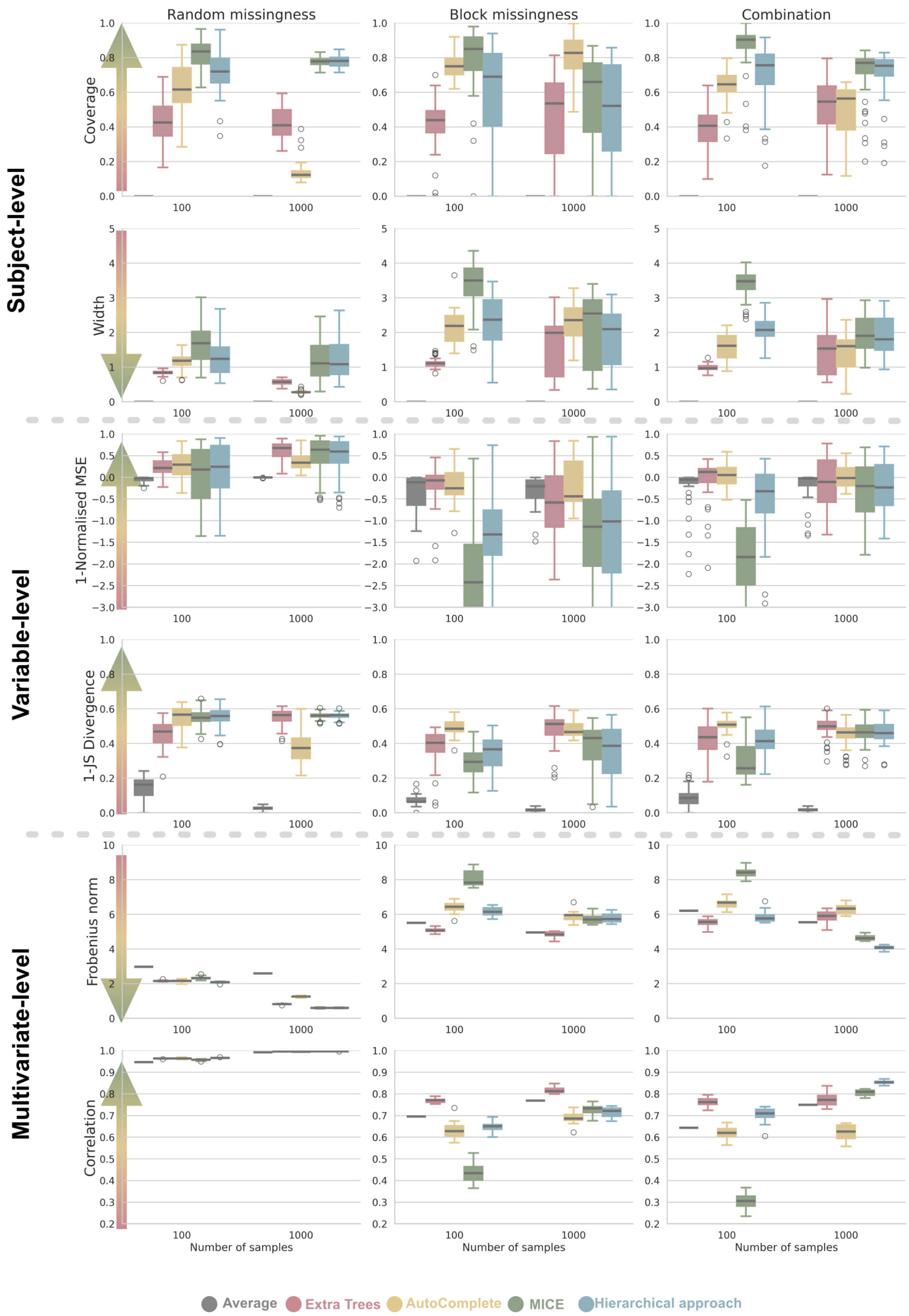

Figure S5: Evaluation of the imputation methods on the simulated datasets: Each row represents a quality of imputation criterion, and each column represents a different type of missingness. The results are shown for five imputation algorithms: Average imputation, Extra Trees, AutoComplete, MICE, and hierarchical MICE; and for two different dataset sizes (100, 1000). Here, the amount of random noise missingness was set to 30%, while both batteries overlapped in 10 variables. The arrows on the left side point in the direction of the ideal reconstruction values.

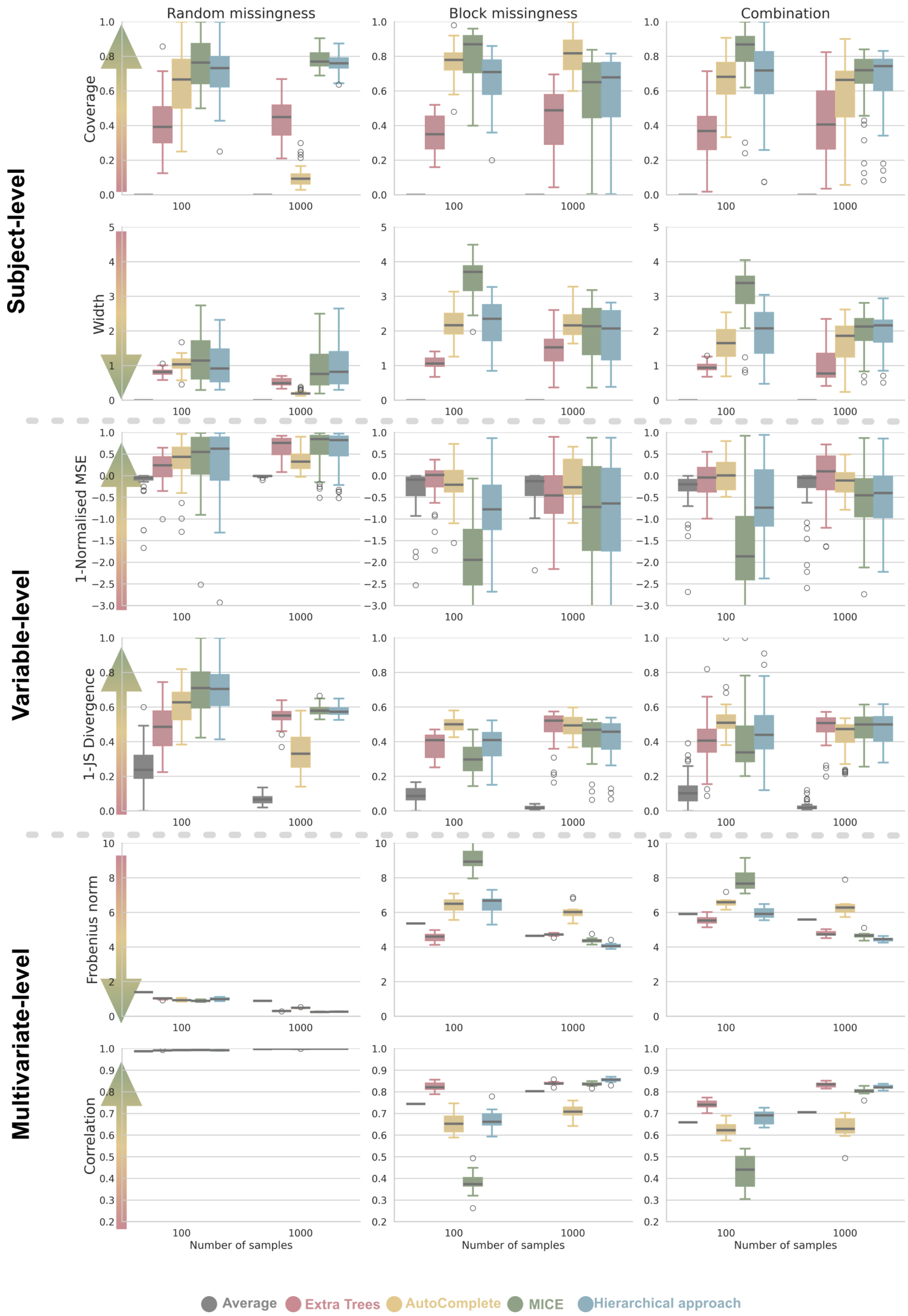

Figure S6: Evaluation of the imputation methods on the simulated datasets: Each row represents a quality of imputation criterion, and each column represents a different type of missingness. The results are shown for five imputation algorithms: Average imputation, Extra Trees, AutoComplete, MICE, and hierarchical MICE; and for two different dataset sizes (100, 1000). Here, the amount of random noise missingness was set to 10%, while both batteries overlapped in 10 variables. The arrows on the left side point in the direction of the ideal reconstruction values.

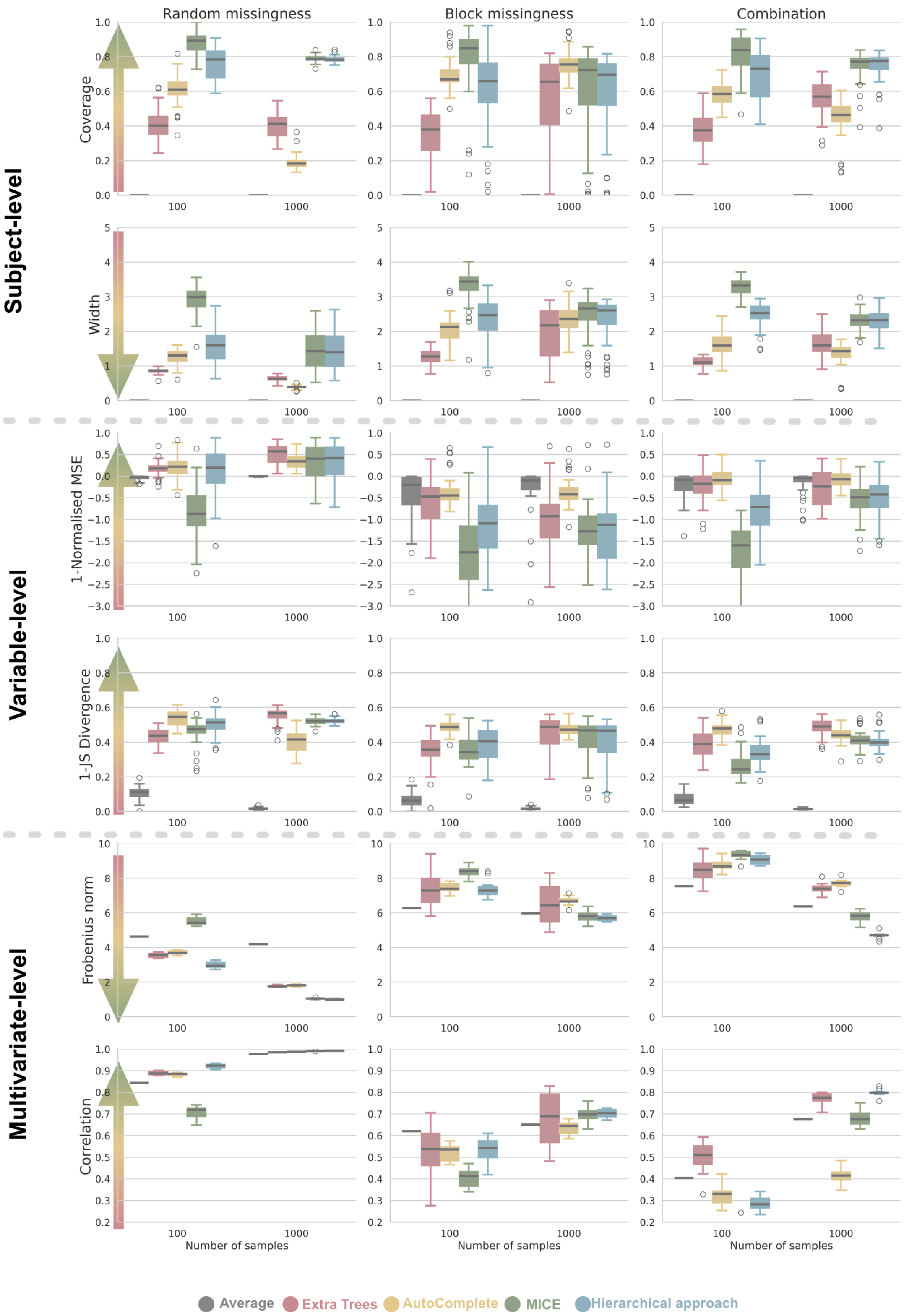

Figure S7: Evaluation of the imputation methods on the simulated datasets: Each row represents a quality of imputation criterion, and each column represents a different type of missingness. The results are shown for five imputation algorithms: Average imputation, Extra Trees, AutoComplete, MICE, and hierarchical MICE; and for two different dataset sizes (100, 1000). Here, the amount of random noise missingness was set to 50%, while both batteries overlapped in 4 variables. The arrows on the left side point in the direction of the ideal reconstruction values.

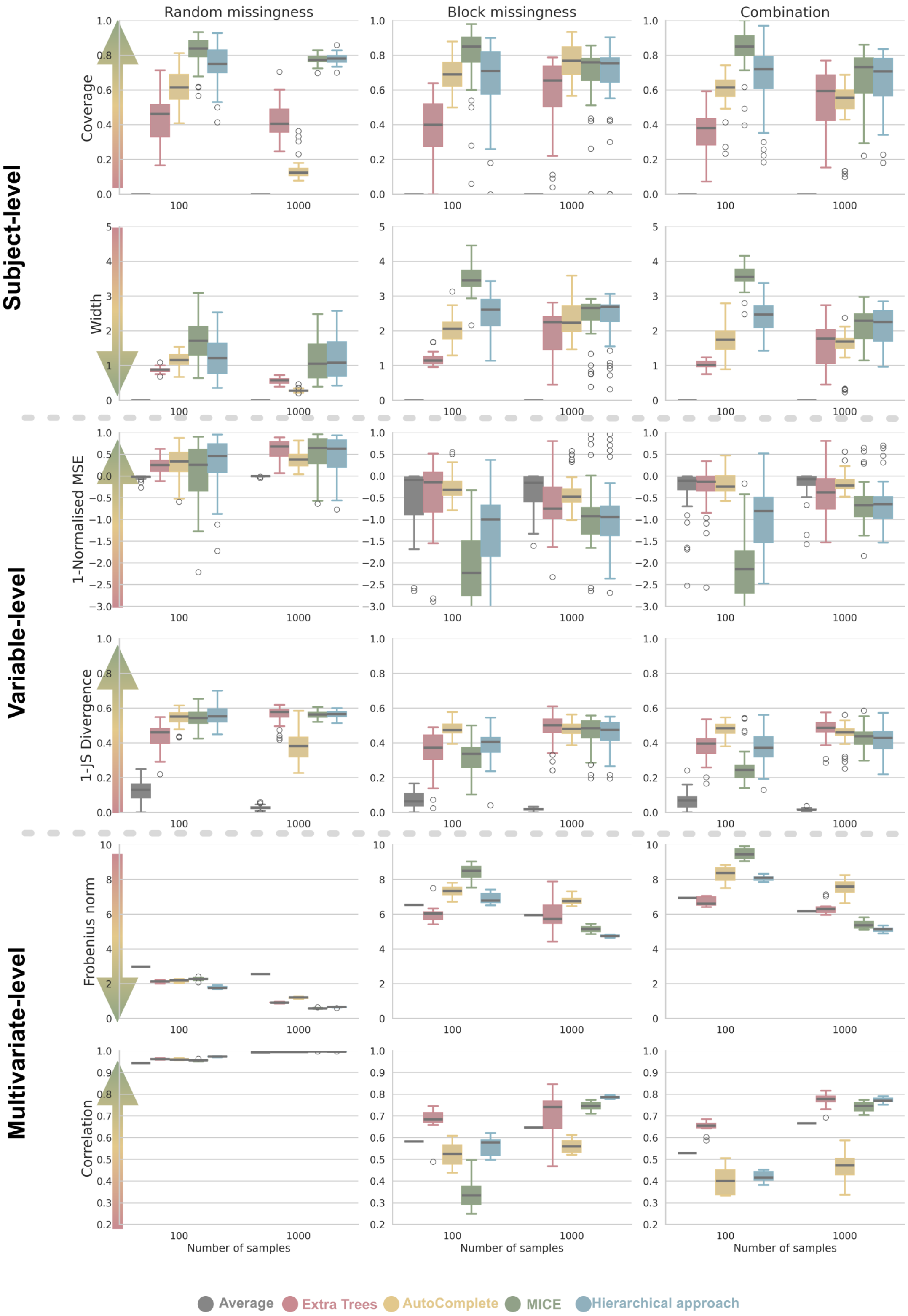

Figure S8: Evaluation of the imputation methods on the simulated datasets: Each row represents a quality of imputation criterion, and each column represents a different type of missingness. The results are shown for five imputation algorithms: Average imputation, Extra Trees, AutoComplete, MICE, and hierarchical MICE; and for two different dataset sizes (100, 1000). Here, the amount of random noise missingness was set to 30%, while both batteries overlapped in 4 variables. The arrows on the left side point in the direction of the ideal reconstruction values.

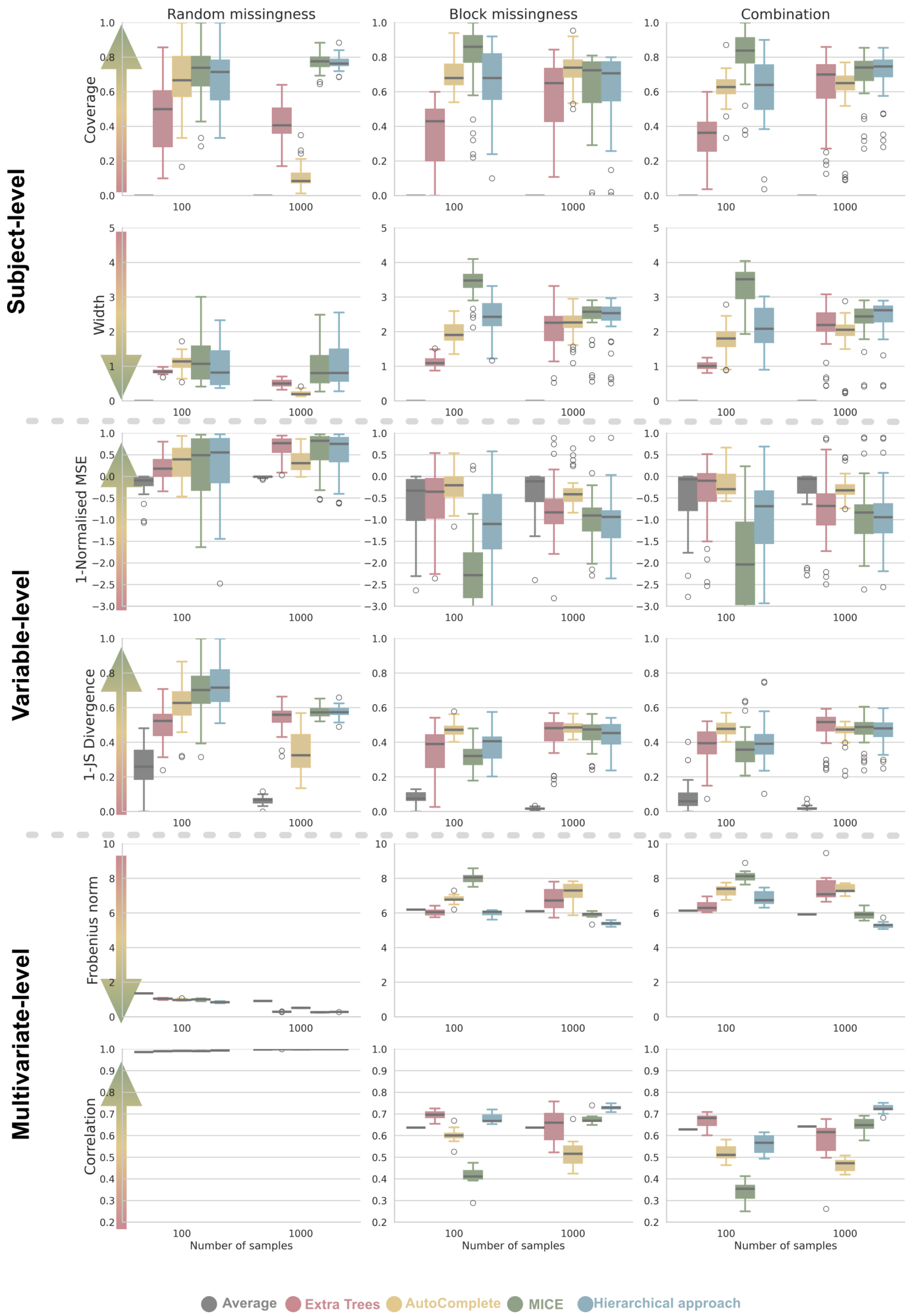

Figure S9: Evaluation of the imputation methods on the simulated datasets: Each row represents a quality of imputation criterion, and each column represents a different type of missingness. The results are shown for five imputation algorithms: Average imputation, Extra Trees, AutoComplete, MICE, and hierarchical MICE; and for two different dataset sizes (100, 1000). Here, the amount of random noise missingness was set to 10%, while both batteries overlapped in 4 variables. The arrows on the left side point in the direction of the ideal reconstruction values.

Supplementary Table

Below, we present a table of the cognitive tests measured in all seven tested cohorts.

Table S1: Cognitive tests. Abbreviations: CANTAB – Cambridge Neuropsychological Test Automated Battery; MCCB – The MATRICS™ Consensus Cognitive Battery; WASI; WAIS3; CVLT; HVLT; WMS – Wechsler Memory Scale; D-KEFS Color Word Interference tests

Battery	Test	Variable	TOP1	TOP2	CNSR 1	CNSR 2	CNSR 3
CANTAB	Delayed Matching to Sample	Correct latency (all delays)				3579.75, 999.86 (1774.66–6196.14)	
CANTAB	Delayed Matching to Sample	Correct				36.70, 5.16 (0.00–40.00)	
CANTAB	Delayed Matching to Sample	Correct (all delays)				27.08, 4.00 (0.00–30.00)	27.38, 2.22 (21.00–30.00)
CANTAB	Delayed Matching to Sample	Correct (simulataneous)				9.62, 1.38 (0.00–10.00)	
CANTAB	Emotion Recognition Task	Mean Overall Response Latency				1391.45, 405.91 (797.12–2618.21)	
CANTAB	Emotion Recognition Task	Percent Correct				70.31, 6.30 (47.22–80.56)	
CANTAB	Intra-Extra Dimensional Set Shift	Errors made at the Extradimensional Shift stage dvs. Stage 8			3.29, 4.50 (0.00–27.00)	6.61, 8.14 (0.00–30.00)	7.10, 9.08 (0.00–32.00)
CANTAB	Intra-Extra Dimensional Set Shift	Completed Stage Trials (number of trials/responses on all successfully completed trials)			70.05, 8.90 (46.00–98.00)	67.69, 10.62 (45.00–112.00)	
CANTAB	Intra-Extra Dimensional Set Shift	Total Errors (adjusted for stages not completed, i.e. 25 pr stage)			12.30, 9.09 (6.00–57.00)	16.61, 15.73 (5.00–63.00)	17.54, 16.65 (6.00–62.00)
CANTAB	Paired Associates Learning	First Trial Memory Score				23.84, 2.24 (17.00–26.00)	
CANTAB	Paired Associates Learning	PAL Total Errors (6 shapes)				0.76, 1.35 (0.00–6.00)	
CANTAB	Paired Associates Learning	PAL Total Errors (8 shapes)				1.62, 2.11 (0.00–8.00)	
CANTAB	Reaction Time	Choice Movement Time			414.85, 88.47 (240.88–709.38)	370.49, 110.65 (193.86–754.25)	395.15, 55.08 (284.62–522.00)
CANTAB	Reaction Time	Choice Reaction Time			327.59, 38.56 (221.25–428.25)	318.20, 44.13 (234.00–467.00)	317.77, 37.38 (253.75–444.14)
CANTAB	Reaction Time	Movement Time			422.97, 91.13 (216.33–647.44)	385.90, 125.56 (197.11–835.11)	404.40, 59.21 (263.11–533.44)
CANTAB	Reaction Time	Reaction Time			304.89, 40.08 (221.33–408.78)	292.37, 41.09 (215.78–484.38)	293.18, 38.25 (238.50–397.56)
CANTAB	Rapid Visual Information Processing	Signal Detection Measure of Sensitivity to Errors Test 3-5-7			0.99, 0.01 (0.96–1.00)	0.99, 0.01 (0.92–1.00)	0.99, 0.01 (0.92–1.00)
CANTAB	Rapid Visual Information Processing	Signal Detection Measure of Sensitivity to Errors Test 3-5-7, 2-4-6			0.98, 0.02 (0.91–1.00)	0.97, 0.03 (0.80–1.00)	0.97, 0.03 (0.83–1.00)
CANTAB	Rapid Visual Information Processing	Latency 3-5-7			351.02, 49.52 (243.14–492.31)	338.70, 77.26 (197.58–755.03)	
CANTAB	Rapid Visual Information Processing	Latency 3-5-7, 2-4-6			377.89, 49.86 (272.70–582.05)	371.06, 67.24 (190.05–702.13)	

Table S1: Cognitive tests. Abbreviations: CANTAB – Cambridge Neuropsychological Test Automated Battery; MCCB – The MATRICS™ Consensus Cognitive Battery; WASI; WAIS3; CVLT; HVLT; WMS – Wechsler Memory Scale; D-KEFS Color Word Interference tests

Battery	Test	Variable	TOP1	TOP2	CNSR 1	CNSR 2	CNSR 3
CANTAB	Rapid Visual Information Processing	Latency 3-5-7, 2-4-6, 4-6-8				368.02, 56.13 (270.56–538.30)	
CANTAB	Stockings of Cambridge	Mean Initial Thinking Time (5 Moves)			9347.43, 7443.26 (1328.75–42198.25)	12382.92, 7398.09 (2244.00–46517.00)	
CANTAB	Stockings of Cambridge	Mean Number of Moves (5 Moves)			6.10, 1.40 (5.00–12.00)	5.79, 0.92 (5.00–8.50)	
CANTAB	Stockings of Cambridge	Mean Subsequent Thinking Time (5 Moves)			649.71, 1085.93 (0.00–5041.30)	392.59, 763.86 (0.00–4125.00)	
CANTAB	Stockings of Cambridge	Mean Subsequent Thinking Time (All Moves)			508.23, 726.96 (0.00–3730.13)	122.74, 241.83 (0.00–921.25)	621.58, 864.19 (0.00–3051.74)
CANTAB	Stockings of Cambridge	Mean Initial Thinking Time (All Moves)			5415.58, 3244.37 (812.88–16795.50)	1335.53, 841.28 (0.00–4428.50)	9929.79, 6167.35 (104.75–30802.00)
CANTAB	Stockings of Cambridge	Mean Number of Moves (All Moves)			4.05, 0.52 (3.50–6.13)		
CANTAB	Stockings of Cambridge	Problems Solved in Minimum Moves			9.54, 2.04 (5.00–12.00)	10.18, 1.36 (6.00–12.00)	9.94, 1.46 (6.00–12.00)
CANTAB	Spatial Working Memory	Strategy			26.49, 5.59 (18.00–42.00)	24.89, 5.77 (18.00–39.00)	23.86, 4.76 (18.00–35.00)
CVLT /BACS		Test Total Number Correct (5 Trials) / Verbal memory Total Recall	56.74, 9.41 (25.00–78.00)			57.22, 8.02 (36.00–72.00)	54.00, 7.43 (31.00–74.00)
CVLT		Free Recall	15.97, 0.20 (14.00–16.00)				
CVLT /HVLT		Long Delay Free Recall /16 / Delayed Recall /12	0.82, 0.16 (0.12–1.00)	0.86, 0.14 (0.42–1.00)			
CVLT /HVLT		Recognition Hits/16 / RecognitionHits/12	0.96, 0.07 (0.50–1.00)	0.98, 0.04 (0.58–1.00)			
DKEFS	Color Word Interference	Inhibition	49.54, 10.10 (30.00–107.00)	47.81, 9.40 (10.00–79.30)			
DKEFS	Color Word Interference	Switching	55.53, 12.25 (1.00–122.00)	54.36, 10.99 (30.00–131.00)			
DKEFS	Verbal Fluency	Semantic	48.95, 8.12 (23.00–76.00)				
DKEFS	Verbal Fluency	Phonemic	45.33, 10.98 (17.00–74.00)				
	Grooved Pegboard	Dominant side (s)	62.37, 9.34 (26.00–116.00)	59.03, 7.83 (41.00–93.00)			
	Grooved Pegboard	Non-dominant side (s)	69.32, 10.95 (8.00–116.00)	64.89, 9.45 (1.09–104.00)			
	Hopkins verbal learning test revised	Total		28.57, 3.96 (17.00–36.00)			

Table S1: Cognitive tests. Abbreviations: CANTAB – Cambridge Neuropsychological Test Automated Battery; MCCB – The MATRICS™ Consensus Cognitive Battery; WASI; WAIS3; CVLT; HVLT; WMS – Wechsler Memory Scale; D-KEFS Color Word Interference tests

**Battery**	**Test**	**Variable**	**TOP1**	**TOP2**	**CNSR 1**	**CNSR 2**	**CNSR 3**
MATRICS		Mazes		22.29, 4.19 (3.00–58.00)			
	Brief Visual Memory Test		22.54, 5.80 (6.50–34.00)	27.88, 5.28 (8.00–37.00)			
	Rey Complex Figure Test	Copying	35.19, 1.26 (29.00–36.00)				
		Trailmaking test Part A (s)		26.40, 8.23 (11.00–65.00)			
	University of California Performance Based Skills Assessment-Brief version	Total Score		86.45, 8.27 (64.00–100.00)			
MCCB /BACS	Executive Functioning	(MCCB) Semantic Verbal Fluency / (BACS) Animal Naming Fluency	48.95, 8.12 (23.00–76.00)	28.89, 6.20 (14.00–55.00)	25.51, 5.29 (13.00–41.00)		
WAIS3 /BACS			0.64, 0.11 (0.33–0.96)	0.65, 0.10 (0.34–1.22)			
WAIS3		Digit Span	16.25, 3.49 (7.00–27.00)	16.93, 3.79 (7.00–28.00)			
WAIS3		Digit Span Backwards	5.00, 1.18 (2.00–8.00)	5.07, 1.35 (2.00–9.00)			
WAIS3		Digit Span Forward	6.25, 1.13 (4.00–9.00)	6.52, 1.26 (4.00–15.00)			
WAIS3	Digit Symbol Coding	Total Number of Digits / 90	0.86, 0.15 (0.44–1.28)				
WAIS3 /MATRICS	Letter Number Sequence		11.44, 2.50 (4.00–18.00)	15.62, 3.09 (6.00–41.00)			
WASI /WAIS 3	Block Design		53.67, 11.46 (5.00–71.00)	55.11, 11.28 (6.00–71.00)		53.78, 8.42 (24.00–67.00)	54.56, 10.33 (30.00–68.00)
WASI /WAIS 3	Matrix Reasoning		28.15, 3.70 (9.00–35.00)	29.20, 3.39 (9.00–50.00)		21.98, 2.59 (15.00–26.00)	21.64, 2.78 (14.00–26.00)
WASI /WAIS 3	Similarities		39.12, 4.24 (19.00–48.00)	38.08, 4.24 (16.00–47.00)		25.33, 4.23 (11.00–32.00)	24.96, 3.86 (14.00–32.00)
WASI /WAIS 3	Vocabulary		61.96, 7.08 (36.00–79.00)	63.41, 7.23 (22.00–77.00)		37.19, 8.93 (19.00–60.00)	37.52, 6.78 (21.00–58.00)
	Wisconsin Card Sorting Test	Failure to Maintain Set	0.42, 0.64 (0.00–3.00)				
	Wisconsin Card Sorting Test	Number of Categories	3.63, 1.26 (0.00–5.00)				
	Wisconsin Card Sorting Test	Preservative Error	6.99, 3.92 (2.00–28.00)				
	Wisconsin Card Sorting Test	Preservative Responses	7.53, 4.71 (2.00–33.00)				

Table S1: Cognitive tests. Abbreviations: CANTAB – Cambridge Neuropsychological Test Automated Battery; MCCB – The MATRICS™ Consensus Cognitive Battery; WASI; WAIS3; CVLT; HVLT; WMS – Wechsler Memory Scale; D-KEFS Color Word Interference tests

Battery	Test	Variable	TOP1	TOP2	CNSR 1	CNSR 2	CNSR 3
	Wechsler Memory Scale Logical Memory	Free Recall	23.80, 6.61 (4.00–42.00)				
	Wechsler Memory Scale Logical Memory	Immediate Story Recall A (Total number items freely recalled after 30)	14.24, 3.62 (4.00–23.00)	15.32, 3.65 (4.00–25.00)			
	Wechsler Memory Scale Logical Memory	Immediate Story Recall B (Total number items freely recalled after 30)	12.40, 3.30 (2.00–22.00)	13.65, 3.73 (5.00–23.00)			
	Wechsler Memory Scale Logical Memory	Immediate Thematic Recall A (Total number items freely recalled after 30)	5.51, 2.60 (1.00–56.00)	5.80, 1.30 (1.00–8.00)			
	Wechsler Memory Scale Logical Memory	Immediate Thematic Recall B (Total number items freely recalled after 30)	5.69, 1.38 (2.00–8.00)	5.93, 1.31 (1.00–8.00)			
	Wechsler Memory Scale Logical Memory	Total Recall	26.60, 6.12 (6.00–45.00)	28.95, 6.52 (11.00–45.00)			

Table S1: Descriptive statistics (mean, standard deviation, and range) for cognitive test variables across datasets: TOP1, TOP2, CNSR1, CNSR2, and CNSR3. Variables reflect various cognitive domains such as memory, attention, emotion recognition, executive function, and processing speed. Empty cells indicate missing data for that particular dataset and variable.
